# Activation of CD44/PAK1/AKT signaling promotes resistance to FGFR1 inhibition in squamous-cell lung cancer

**DOI:** 10.1038/s41698-022-00296-2

**Published:** 2022-07-19

**Authors:** Omar Elakad, Björn Häupl, Vera Labitzky, Sha Yao, Stefan Küffer, Alexander von Hammerstein-Equord, Bernhard C. Danner, Manfred Jücker, Henning Urlaub, Tobias Lange, Philipp Ströbel, Thomas Oellerich, Hanibal Bohnenberger

**Affiliations:** 1 Institute of Pathology, University Medical Center, Goettingen, Germany; 2 Department of Medicine II, Hematology/Oncology, Goethe University, Frankfurt, Germany; 3 German Cancer Research Center and German Cancer Consortium, Heidelberg, Germany; 4 Frankfurt Cancer Institute, Goethe University, Frankfurt, Germany; 5 Institute for Anatomy and Experimental Morphology, University Cancer Center, University Medical Center Hamburg-Eppendorf, Hamburg, Germany; 6 Department of Thoracic and Cardiovascular Surgery, University Medical Center, Goettingen, Germany; 7 Center for Experimental Medicine, Institute of Biochemistry and Signal Transduction, University Medical Center Hamburg-Eppendorf, Hamburg, Germany; 8 Bioanalytical Mass Spectrometry Group, Max Planck Institute for Biophysical Chemistry, Goettingen, Germany

**Keywords:** Cancer therapeutic resistance, Non-small-cell lung cancer

## Abstract

Lung cancer is the leading cause of cancer-related deaths worldwide. Fibroblast growth factor receptor 1 (*FGFR1*) gene amplification is one of the most prominent and potentially targetable genetic alterations in squamous-cell lung cancer (SQCLC). Highly selective tyrosine kinase inhibitors have been developed to target FGFR1; however, resistance mechanisms originally existing in patients or acquired during treatment have so far led to limited treatment efficiency in clinical trials. In this study we performed a wide-scale phosphoproteomic mass-spectrometry analysis to explore signaling pathways that lead to resistance toward FGFR1 inhibition in lung cancer cells that display (i) intrinsic, (ii) pharmacologically induced and (iii) mutationally induced resistance. Additionally, we correlated AKT activation to CD44 expression in 175 lung cancer patient samples. We identified a CD44/PAK1/AKT signaling axis as a commonly occurring resistance mechanism to FGFR1 inhibition in lung cancer. Co-inhibition of AKT/FGFR1, CD44/FGFR1 or PAK1/FGFR1 sensitized ‘intrinsically resistant’ and ‘induced-resistant’ lung-cancer cells synergetically to FGFR1 inhibition. Furthermore, strong CD44 expression was significantly correlated with AKT activation in SQCLC patients. Collectively, our phosphoproteomic analysis of lung-cancer cells resistant to FGFR1 inhibitor provides a large data library of resistance-associated phosphorylation patterns and leads to the proposal of a common resistance pathway comprising CD44, PAK1 and AKT activation. Examination of CD44/PAK1/AKT activation could help to predict response to FGFR1 inhibition. Moreover, combination between AKT and FGFR1 inhibitors may pave the way for an effective therapy of patients with treatment-resistant *FGFR1*-dependent lung cancer.

## Introduction

Lung cancer is the most frequent cancer type, both in incidence (worldwide, 11.6% of all cancers in 2018) and in cancer-related mortality (18.4% in 2018)^1^. Histologically, it is classified into two major groups: small-cell lung cancer (SCLC, ~15%) and non-small-cell lung cancer (NSCLC, ~85%); the latter includes the subtypes adenocarcinoma (AC, ~40%) and squamous-cell lung cancer (SQCLC, ~30%)^2^.

Molecular characterization of lung cancer opened the door for the development of molecule-targeted treatments such as tyrosine kinase inhibitors (TKIs)^3,4^. There have been reports of well-documented success from the use of TKIs as a first-line treatment for pulmonary adenocarcinoma patients who harbor *EGFR* mutations or ALK translocations, e.g., with Gefitinib/Erlotinib and Crizotinib, respectively^5^. In contrast, squamous-cell and small-cell lung cancers lag behind adenocarcinoma, with very few FDA-approved targeted therapies, e.g., PDL1 monoclonal antibodies for metastatic SQCLC^6,7^.

Fibroblast growth factor receptor 1 (*FGFR1*) gene amplification is a common gene alteration in SQCLC (encountered in 16–23% of cases) and SCLC (5.6–8%); hence, it might lead to a molecular-therapeutic option for these groups^8–10^. Amplification of the *FGFR1* gene has also been reported in other tumor entities, such as breast, colon and pancreatic cancers^11–13^.

Highly specific compounds have been developed to inhibit FGFR1 by suppressing its kinase activity (e.g., AZD4547 and BGJ398)^14^. Clinical trials in phases I and II have proven the safety and activity of these specific inhibitors in SQCLC, with a disease-control rate of 26–39%; some patients have profited from the treatment for more than 14 months^15–17^. Nevertheless, a considerable group of patients showed immediate or acquired resistance to the treatment. The ability of SQCLC cells to escape FGFR1 inhibition is probably due to compensation mechanisms that either originally existed in these cancer cells (intrinsic resistance) or developed over the course of treatment (induced resistance)^18,19^. Achieving a sustainable response to FGFR1 inhibition depends on the ability to identify and describe accurately these resistance mechanisms and to find new targets that can be combined with FGFR1 inhibition.

In the present project, we aimed to investigate resistance mechanisms to FGFR1 inhibition in lung-cancer cells with *FGFR1* gene amplification. Our phosphoproteomic mass-spectrometric analysis revealed a common resistance pathway comprising activation of CD44, Pak1 and AKT. Co-inhibition of CD44, Pak1 or AKT together with FGFR1 (re-)sensitized resistant lung cancer cells to *FGFR1*-targeted therapy.

## Results

### Phosphoproteomic LC-MS/MS analysis reveals activation of AKT as an intrinsic resistance mechanism against FGFR1 inhibition

**Fig. 1:**
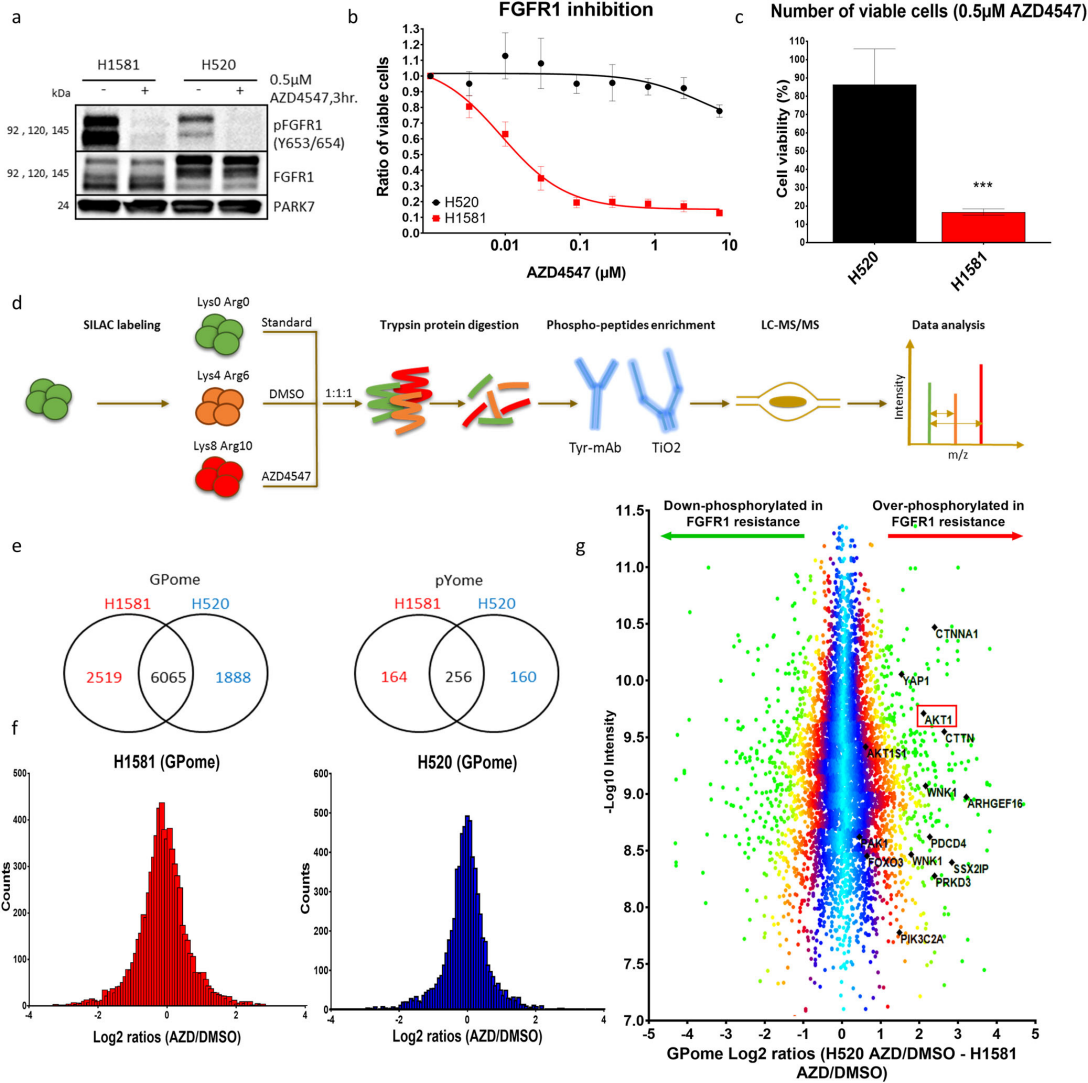
Differential phosphoproteomic analysis between cells that are sensitive or resistant to FGFR1 inhibition. **a** Western blot showing expression and phosphorylation of FGFR1 protein under DMSO and AZD4547 treatment. **b** Cell viability analysis of the two lung-cancer cell lines NCI-H1581 and NCI-H520 after treatment with increasing concentrations of the FGFR1 inhibitor AZD4547 for 96 h. **c** Viable cells were counted after treatment with 0.5 µM AZD4547 for 96 h and compared with control cells treated with DMSO. **d** Workflow of SILAC labeling, phosphopeptide enrichment and LC-MS/MS analysis. **e**, **f** Numeric Venn diagram of phosphosites counted in GPome and pYome and the normal distribution of SILAC ratios between the two cell lines NCI-H1581 (**e**) and NCI-H520 (**f**). **g** Over- and down-phosphorylated sites in the FGFR1-inhibition-resistant cell line NCI-H520 compared with the sensitive cell line H1581 under AZD4547 treatment controlled against DMSO treatment. Statistical analysis was performed with the chi-squared test: ns (*p* > 0.05), *(*p* < 0.05), **(*p* ≤ 0.01) and ***(*p* ≤ 0.001). Mean values are plotted; error bars represent standard deviation.

In order to study different mechanisms of resistance to FGFR1 inhibition in lung cancer cells, we compared the two lung cancer cell lines NCI-H1581 and NCI-H520, both of which harbor an *FGFR1* gene amplification and a corresponding overexpression of FGFR1 RNA and protein (Fig. 1a and S1a, b). Despite effective inhibition of FGFR1 phosphorylation (Fig. 1a), NCI-H1581 and NCI-H520 cells showed opposite responses to their respective specific FGFR1 inhibitors (AZD4547 and BGJ398), a result that was validated by knockdown of FGFR1 expression by directed interfering RNAs (siRNAs) (Figs. 1b, c and S1c–f). These results showed that NCI-H1581 has a high sensitivity to FGFR1 inhibition, with IC50 values of 30–80 nM, compared with >5 µM in the resistant cell line NCI-H520.

In order to elucidate the difference in signaling status between FGFR1 inhibition-sensitive and inhibition-resistant cell lines, we performed a quantitative phosphoproteomic mass-spectrometric comparison (LC-MS/MS) of the sensitive NCI-H1581 and the resistant NCI-H520 cell lines under treatment with DMSO and AZD4547. For stable-isotope labeling with heavy amino acids in cell culture (SILAC), both cell lines were cultured with either ‘medium-heavy’ or ‘heavy’ isotopically labelled arginine and lysine, for at least ten cell cycles to ensure full incorporation of heavy amino acids. DMSO was used to treat ‘medium-heavy’ SILAC-labelled cells, while ‘heavy’ SILAC-labelled cells were treated with 0.5 µM AZD4547 (FGFR1 inhibitor) for 3 h before cell lysis. To allow accurate comparison between sensitive and resistant cells under different conditions, we also included a protein quantification standard (spike-in SILAC standard), containing a mixture of cells (four lung-cancer cell lines) in equal amounts (NCI-H1581, DMS114, NCI-H520 and NCI-H1703) cultured in ‘light’ growth medium. Titanium dioxide was used to enrich the mixture in phosphorylated peptides, which are mainly serine and threonine peptides and to a lesser extent tyrosine-containing peptides (GPome analysis). In addition, enrichment in tyrosine-phosphorylated peptides was carried out by using specific tyrosine antibodies (pYome analysis) before LC-MS/MS analysis (Fig. 1d).

We were able to quantify 10,472 (8913 class I) phosphosites in our GPome analysis and 580 (503 class I) phosphosites in our pYome analysis, with normal distribution of log_2_ SILAC ratios in both cell lines (Fig. 1e, f and Supplementary Table 1). The distribution of phosphosites was 89.4%, 9.8% and 0.8% of the serine, threonine and tyrosine sites (respectively) in the GPome group. By comparing the ratios of protein phosphorylation in AZD4547-treated cells and DMSO-treated cells in resistant (NCI-H520) and sensitive (NCI-H1581) cell lines, we found strong and significant phosphorylation of AKT1 at the activation-site serine-124 and its downstream target PRAS40 (AKT1S1) at threonine-246 in the resistant cell line NCI-H520 compared with the sensitive cell line H1581 (Fig. 1g and Supplementary Table 2). Moreover, several proteins related to AKT activation – e.g., FOXO3, PIK3C, YAP1, CTTN, WNK1 and CTNNA1 – were found to be overphosphorylated in the resistant cell line.

**Fig. 2:**
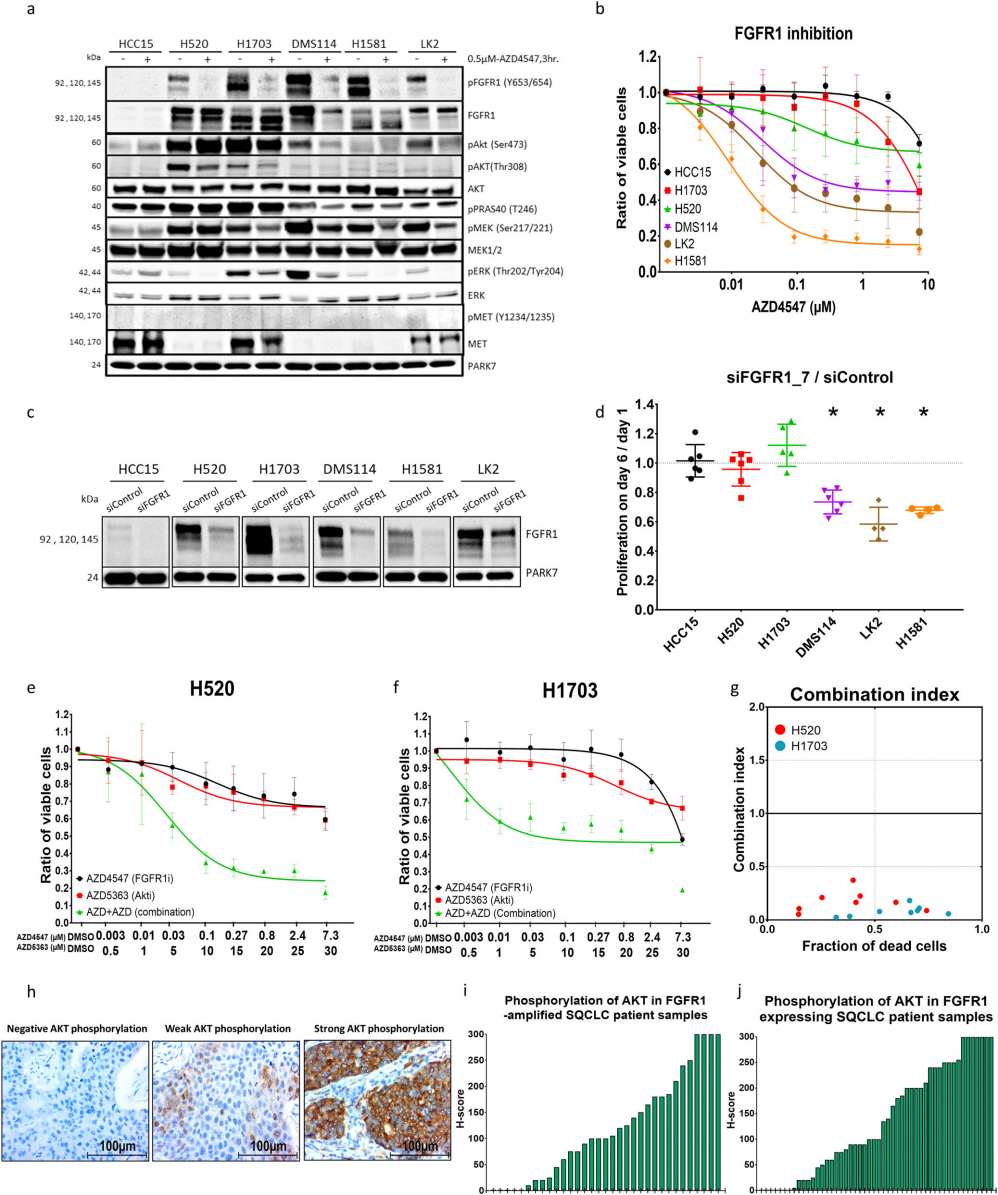
Intrinsic AKT activation induces FGFR1 inhibition resistance in lung-cancer cells. **a** Western blot analysis comparing main signaling pathways between control (HCC15), resistant (NCI-H520 and NCI-H1703) and sensitive (DMS114, H1581 and LK2) lung-cancer cell lines after FGFR1 inhibition with AZD4547. **b** Cell viability assay comparing sensitivity profiles of the six lung-cancer cell lines indicated to FGFR1 inhibition with DMSO treatment as standard. **c**, **d** Knockdown of FGFR1 with small interfering RNA (siRNA, SI02224684) in different lung-cancer cell lines validated by western blot analysis after 48 h (**c**) and proliferation of cells was compared on day 6 (**d**). **e**, **f** Cell viability assay showing the effect of combining the AKT inhibitor AZD5363 with the FGFR1 inhibitor AZD4547 at different concentrations after 96 h incubation in NCI-H520 (**e**) and NCI-H1703 (**f**) SQCLC cell lines. **g** Combination index plot showing the synergetic effect of combining AZD4547 with AZD5363 in NCI-H520 and NCI-H1703 cell lines calculated using CompuSyn software based on the Chou–Talalay drug interaction algorithm: CI < 1, synergetic effect; CI = 1, additive effect; CI > 1, antagonistic effect. **h** Immunohistochemical staining of SQCLC patient tissue samples with negative, weak and strong phosphorylated AKT signals. **i**, **j** H-score quantification of pAKT signals in the SQCLC patients with FGFR1 amplification (**i**) and in the SQCLC patients with FGFR1 overexpression (**j**). Statistical analysis was performed with the chi-squared test: ns (*p* > 0.05), *(*p* < 0.05), **(*p* ≤ 0.01) and ***(*p* ≤ 0.001). Mean values are plotted; error bars represent standard deviation.

In order to investigate the correlation between AKT activation and resistance to FGFR1 inhibition developed within lung cancer cells, we expanded the investigation to include a total of six lung-cancer cell lines, five of which (NCI-H1581, DMS114, LK2, NCI-H520 and NCI-H1703) showed elevated levels of FGFR1 protein and RNA expression compared with the control cell line HCC15 (Figs. 2a and S1a, b). Fluorescence in situ hybridization analysis revealed an amplification of the *FGFR1* gene locus in NCI-H1581, DMS114, NCI-H520 and NCI-H1703 cell lines but not in LK2 and HCC15 (Fig. S1a). Sensitivity profiles to FGFR1 inhibition were screened and validated by comparing the viability and cell count of viable cells of the six cell lines after treatment with the two FGFR1-specific inhibitors AZD4547 and BGJ398 and knockdown of FGFR1 using two validated siRNA sequences. FGFR1 inhibition significantly decreased cell proliferation in NCI-H1581, DMS114 and LK2 compared with NCI-H520, NCI-H1703 and HCC15, none of which showed any response to the inhibition, despite effective inhibition of FGFR1 phosphorylation after treatment with the FGFR1 inhibitor AZD4547 in all of the FGFR1-expressing cell lines (Fig. 2a–d and S1c–f).

Further western blot analysis confirmed the elevated phosphorylation of AKT at both of its activating phosphorylation sites, serine-473 and threonine-308, within the two cell lines that were resistant to FGFR1 inhibition (NCI-H520 and NCI-H1703), compared with the three cell lines that were sensitive to FGFR1 inhibition (DMS114, NCI-H1581 and LK2), as was also observed in the phosphoproteomic analysis. Notably, the phosphorylation level of AKT in the most sensitive cell line was so low that we have investigated the decrease after FGFR1 inhibition on a separate western blot (Fig. S2a). In addition PRAS40 (also named AKT1S1), the direct downstream target of AKT, was strongly phosphorylated within the resistant cell lines as well (Fig. 2a).

Next, we used western blotting to investigate the concentration at which the AKT inhibitor (AZD5363) blocks the kinase activity of AKT and the activation of its downstream effector (PRAS40). The analysis showed that from an AZD5363 concentration of 1 µM AKT phosphorylation was significantly higher than in untreated cells. However, the phosphorylation levels of its downstream effector (PRAS40) were significantly reduced, with complete disappearance reached at 30 µM (Fig. S2b). The effect of AKT inhibition on AKT overphosphorylation has already been reported^20^. We inhibited pharmacologically both FGFR1 (AZD4547) and AKT (AZD5363) and found a strong reduction in cell viability, particularly in the resistant cells NCI-H520 and NCI-H1703 (Figs. 2e, f, and S2c) with an induction of apoptosis under combination treatment (Fig. S2d). The type of interaction among FGFR1 and AKT inhibitors was explored by using the Chou–Talalay algorithm (CompuSyn), according to the median-effect equation, to elaborate the nature of interaction between two or more inhibitors in the form of combination index values (CI)^21^. Strong synergetic interaction between AZD4547 (FGFR1 inhibitor) and AZD5363 (AKT inhibitor) was shown by the combination index values (Fig. 2g).

Finally, we were able to demonstrate a strong intrinsic variation of AKT phosphorylation by immunohistochemical staining of phosphorylated AKT in tissue samples from patients with squamous-cell lung-cancer characterized by either FGFR1 amplification (*n* = 33) or FGFR1 protein overexpression (*n* = 50) (Fig. 2h–j, Table 1 and Supplementary Table 3).

**Table 1:** Patients’ characteristics and immunohistochemistry staining.

	No. of cases	IHC-pAKT			IHC-CD44		
		Negative/weak	Strong	*p*	Negative/weak	Strong	*p*
Sex							
Male	153	109 (71%)	44 (29%)	0.5445	85 (56%)	67 (44%)	0.3504
Female	30	23 (77%)	7 (23%)		15 (47%)	17 (53%)	
Age							
≤60	49	36 (73%)	13 (27%)	0.8071	22 (45%)	27 (55%)	0.121
>60	134	96 (72%)	38 (28%)		78 (58%)	57 (42%)	
Degree of diffr.							
I + II	138	99 (72%)	39 (28%)	0.8359	69 (50%)	70 (50%)	**0.0315**
III	45	33 (73%)	12 (27%)		30 (68%)	14 (32%)	
LN metastasis							
No	108	72 (67%)	36 (33%)	**0.0479**	61 (60%)	40 (40%)	0.0711
Yes	75	60 (80%)	15 (20%)		33 (46%)	38 (54%)	
Clinical stage							
I + II	132	94 (76%)	29 (24%)	0.0637	69 (53%)	62 (47%)	0.5388
III + IV	51	38 (63%)	22 (37%)		30 (58%)	22 (42%)	
Resection status							
R0	168	119 (71%)	49 (29%)	0.19	89 (54%)	77 (46%)	0.6814
R1 + 2	15	13 (87%)	2 (13%)		10 (59%)	7 (41%)	
IHC-pAKT1							
Negative/weak	132				78	48	**0.0012**
Strong	51				17	32	
IHC-CD44							
Negative/weak	100	78	17	**0.0012**			
Strong	84	48	32				
FISH-FGFR1							
Negative	105	68	37	0.1564	46	57	0.2559
Positive	32	25	7		19	15	
IHC-FGFR1							
Negative/weak	109	81	28	0.1142	61	71	**<0.0001**
Strong	50	31	19		25	3	

Bold values represent significant *p*-value  <  0.05.

*p* values were calculated with the chi-squared test; significant values are indicated in bold type.

­*diffr*. differentiation, *LN* lymph node.

### AKT activation induces resistance to FGFR1 inhibition in formerly sensitive cells

**Fig. 3:**
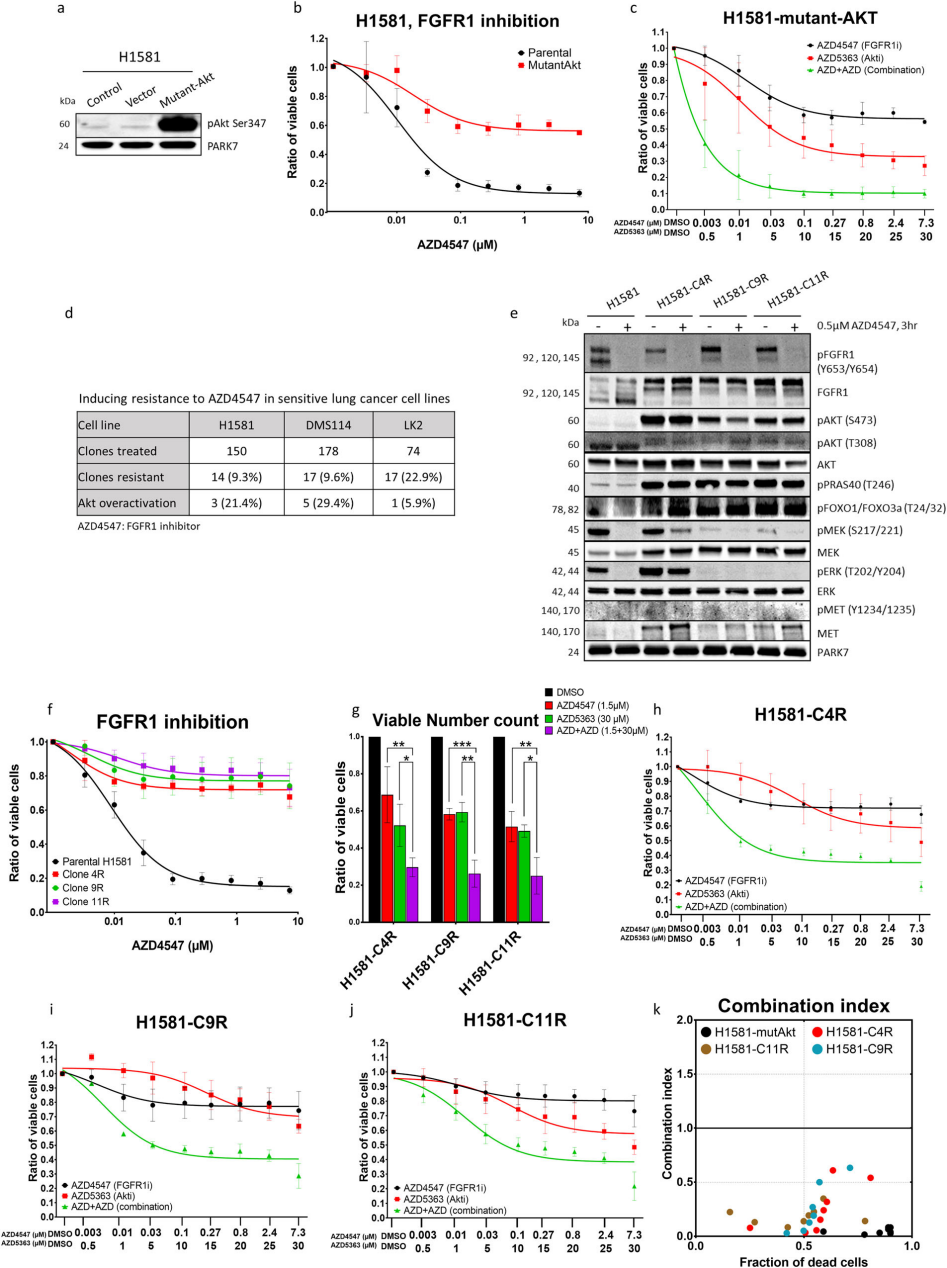
Induced AKT activation promotes FGFR1 inhibition resistance in lung-cancer cells. **a** Western blot analysis showing increase in AKT protein phosphorylation upon transfection with constitutively active AKT mammalian vector. **b**, **c** Cell viability assays showing the effect of single treatment of mutant and parental H1581 cell lines with AZD4547 for 96 h (**b**) or AZD4547 combined with AZD5363 after 96 h (**c**). **d** The table shows numbers and percentages of single cells with induced resistance after continuous treatment with AZD4547 in three lung-cancer cell lines. **e** Western blot analysis comparing FGFR1 and AKT expression and activation among the three induced-resistance single clones H1581-C4R, H1581-C9R and H1581-C11R with their sensitive parental control NCI-H1581 cell line. **f** Cell viability assay measuring the effect of AZD4547 treatment on the three named resistant clones. **g** Counting of viable cells using cell-cycler under treatment of AZD4547 and/or AZD5363. **h**–**j** Cell viability assay for combined AZD4547 and AZD5363 treatments after 96 h in sensitive parental control cell line H1581 and three FGFR1 inhibition-resistant clones H1581-C4R, H1581-C9R and H1581-C11R. **k** Combination index plot calculated with CompuSyn according to the Chou–Talalay equation algorithm: CI < 1, synergetic effect; CI = 1, additive effect; CI > 1, antagonistic effect. Statistical analysis was performed with the chi-squared test: ns (*p* > 0.05), *(*p* < 0.05), **(*p* ≤ 0.01) and ***(*p* ≤ 0.001). Mean values are *p*lotted; error bars represent standard deviation.

After demonstrating the role of AKT in intrinsic resistance to FGFR1 inhibition, we first tested whether activation of AKT is also able to induce resistance to FGFR1 inhibition-sensitive cells. To do this, we transfected the two sensitive cell lines (NCI-H1581 and LK2) with myristoylated AKT (pcDNA3-Myr-AKT1). This led to a strong elevation of AKT phosphorylation as compared with parental (non-transfected) cells or cells transfected with empty vector (Figs. 3a and S2e). Viability assays demonstrated a significant and strong reduction in sensitivity to FGFR1 inhibition on the part of NCI-H1581 and LK2 cells that had been transfected with the mutant-AKT vector. The reduction in sensitivity was successfully reversed by co-inhibition of FGFR1 and AKT. The combination index analysis again revealed a synergetic effect (Figs. 3b, c, k and S2c).

Secondly, we explored whether AKT activation also occurs as an acquired resistance mechanism that evolves after long-term exposure to FGFR1 inhibition. We therefore sorted the three FGFR1 inhibition-sensitive cell lines described above (NCI-H1581, DMS114 and LK2) into single clones. Clones were exposed to repeated cycles of treatment with a high concentration (5 μM) of the FGFR1 inhibitor AZD4547 interrupted by recovery cycles in a process that overall lasted for 10 months.

Resistance to FGFR1 inhibition was achieved in respectively 14 (9.3%), 17 (9.6%) and 17 (22.9%) of the single clones from NCI-H1581, DMS114 and LK2 that had survived the sorting procedure (Fig. 3d). Afterwards, we screened all resistant single clones for phosphorylation of AKT compared with their sensitive parental control cell lines and found that strong AKT phosphorylation was detectable within resistant clones in 21.4% of NCI-H1581, 29.4% of DMS114 and 5.9% of LK2 (Fig. 3d and S3a). Subsequent analysis of the NCI-H1581-derived resistant clones with AKT activation (clones H1581-C4R, H1581-C9R and H1581-C11R) showed significant activation of the AKT downstream effectors PRAS40 and FOXO1/3a, while FGFR1 total protein expression and the inhibitory effect of AZD4547 on FGFR1 phosphorylation were maintained (Fig. 3e). Western blot analysis validated the efficiency of AZD5363 in blocking AKT kinase activity in induced-resistant cells and showed highest activity of AZD5363 at 30 µM (Fig. S3b). Co-inhibition of FGFR1 (AZD4547) and AKT (AZD5363) rescued the response of the resistant cells to FGFR1 inhibition and again demonstrated a strong and synergetic reduction of viability and an induction of apoptosis (Figs. 3f–k and S3c).

### Phosphoproteomic analysis reveals a common upstream signaling pathway leading to AKT activation in FGFR1 inhibition-resistant cells

After identification of AKT activation as a common resistance mechanism in intrinsic and induced resistance to FGFR1 inhibition, we explored the upstream signaling pathways leading to AKT activation. By using DNA- and RNA-directed lung-cancer sequencing panels to compare the three FGFR1 inhibition-resistant single clones with their sensitive parental control cell line (NCI-H1581) we could exclude several known mechanisms of AKT activation, e.g., *AKT, RAS, PTEN, PIK3CA or FGFR1* gatekeeper mutations, overexpression of AKT, FGFR1 or activation of MET (Fig. 3e and S3d, e). Hence, we hypothesized that a mutation-independent mechanism might lead to resistance against FGFR1 inhibitors. Therefore, we analyzed the phosphoproteome of NCI-H1581 as a sensitive parental cell line, NCI-H1581 mutant-AKT as a genetically induced resistant cell line, NCI-H520 as an intrinsically resistant cell line and H1581-C9R and H1581-C11R as induced-resistant cell lines; this was done by mass spectrometry, as described above (Fig. 1d). The inclusion of NCI-H1581 mutant-AKT cell line allowed differentiation between phosphosites that are regulated upstream and downstream of AKT.

**Fig. 4:**
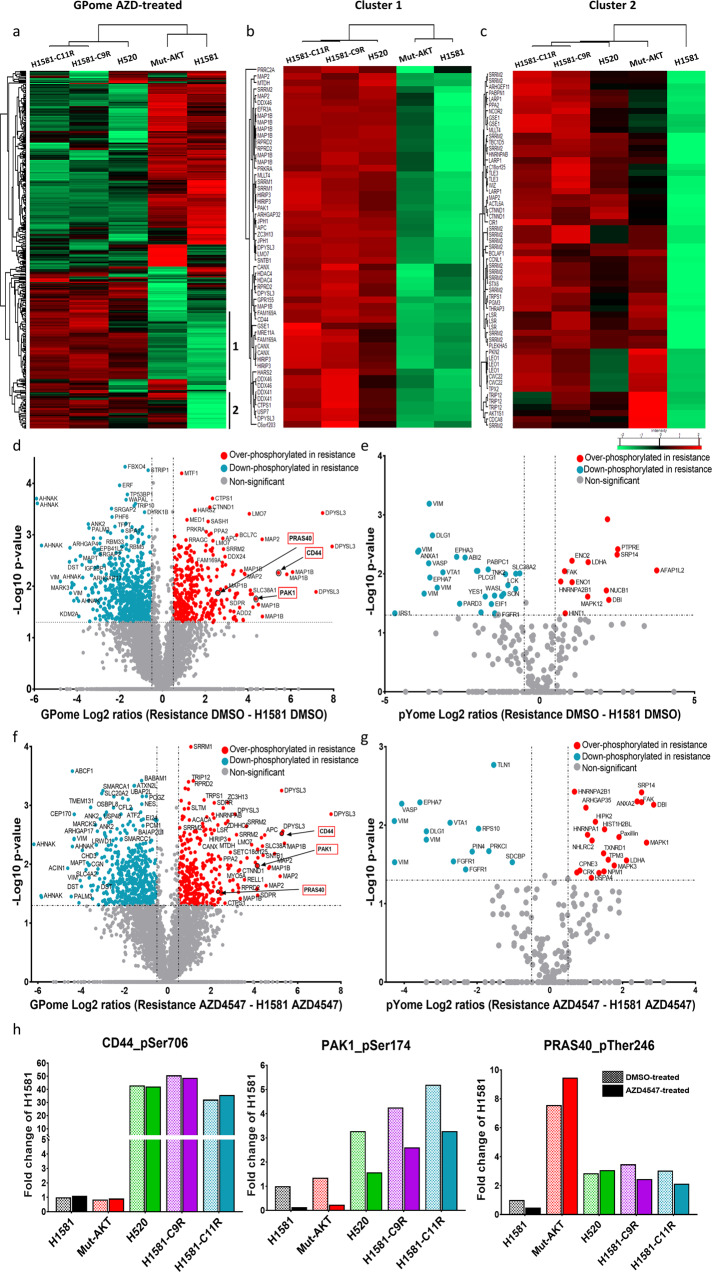
Comprehensive phospho-LC-MS/MS analysis comparing lung-cancer cells that are sensitive to FGFR1 inhibition with those that are resistant. **a**–**c** Heatmaps showing significantly up- and down-regulated phosphorylation in: the native resistant cell line NCI-H520; the H1581 cell line with resistance induced by AKT mutational overexpression; two induced-resistant single clones H1581-C9R and H1581-C11R; and the sensitive parental control cell line NCI-H1581. Cells were treated with the FGFR1-inhibitor AZD4547 (**a**); clusters 1 and 2 are shown enlarged (**b** and **c**). **d**–**g** Volcano plots comparing significantly over- and down-phosphorylated sites according to ANOVA in the sensitive parental control cell line H1581 compared with combined NCI-H520, H1581-C9R and H1581-C11R resistant cell lines after GPome-enrichment treated with DMSO (**d**) or AZD4547 (**f**) after pYome-enrichment treated with DMSO (**e**) or AZD4547 (**g**). **h** Bar charts showing fold change of phosphorylation among different significantly regulated phosphosites within resistant cell lines compared with the sensitive parental control cell line treated with DMSO (H1581) in DMSO-treated (#) and AZD4547-treated cells (*).

A total of 13,893 (11,189 class I) phosphosites were quantified in our GPome analysis and 1011 (866 class I) phosphosites in our pYome analyses (Fig. S4a and Supplementary Table 1). Normal distribution of log_2_ SILAC ratios was observed. Reproducibility was demonstrated with biological (*n* = 2) and technical (*n* = 2) replicates (Figs. S4 and S5). Statistical comparison of DMSO-treated cells by ANOVA revealed 731 and 34 significantly differentially phosphorylated phosphosites after GPome and pYome enrichment, respectively (Fig. S6a). In AZD4547 treated cells, 667 and 86 significantly regulated sites were detected in GPome- and pYome-enriched peptides, respectively (Figs. 4a and S6b). Resulting heatmaps of significantly regulated sites showed a cluster of phosphosites upregulated only in intrinsic and induced FGFR1 inhibition-resistant cells compared with control NCI-H1581 and H1581-mutant AKT cell lines, both in the GPome and the pYome analysis, suggesting that they are regulated upstream of activated AKT (Cluster 1) (Figs. 4a and S6c). These clusters included CD44_S706, PAK1_S174 and AKT1S1_T246, which had already been implicated in AKT regulation (Figs. 4b and S6d)^22,23^. Another cluster of phosphosites found in the GPome analysis was significantly upregulated in the resistant cells, including also the NCI-H1581-mutantAKT, compared with the sensitive parental NCI-H1581 cells, suggesting that these sites are regulated downstream of activated AKT. The later cluster included proteins of known AKT-regulated signaling pathways with functions in apoptosis inhibition (DAXX, ACIN1 and BIM), in proliferation and survival (GAPDH, CTNND1, CTNNB1 and LARP1), and in metastasis (ANXA1 and Cortactin) (Cluster 2) (Fig. 4c)^24–32^.

Combining intrinsically and induced FGFR1 inhibition-resistant cells (NCI-H520, H1581-C9R and H1581-C11R) and comparing them with the parental NCI-H1581-sensitive cells made it possible to identify common phosphosites that are significantly up- and down-regulated in FGFR1 inhibition resistance and may be responsible for its induction. Firstly, we correlated quantified phosphosites from GPome and pYome groups between resistant cell lines and the NCI-H1581 sensitive control cell line under DMSO treatment (Figs. 4d, e, S7a, b and Supplementary Table 2). Secondly, a similar comparison was carried out between resistant and sensitive cells under conditions of FGFR1 inhibition (AZD4547) (Figs. 4f, g, S7c, d and Supplementary Table 2). Inclusion of the AKT-overexpressing NCI-H1581 cell line allowed distinction of regulated phosphorylation sites that are dependent of AKT like e.g. T246 of PRAS40, a well-known downstream target of AK, and phosphorylation sites that are independent and thus potentially upstream of AKT in in resistant cell lines such as S706 of CD44_ and S174 of PAK1 (Fig. 4h).

### CD44 and PAK1 activate AKT and induce resistance to FGFR1 inhibition

**Fig. 5:**
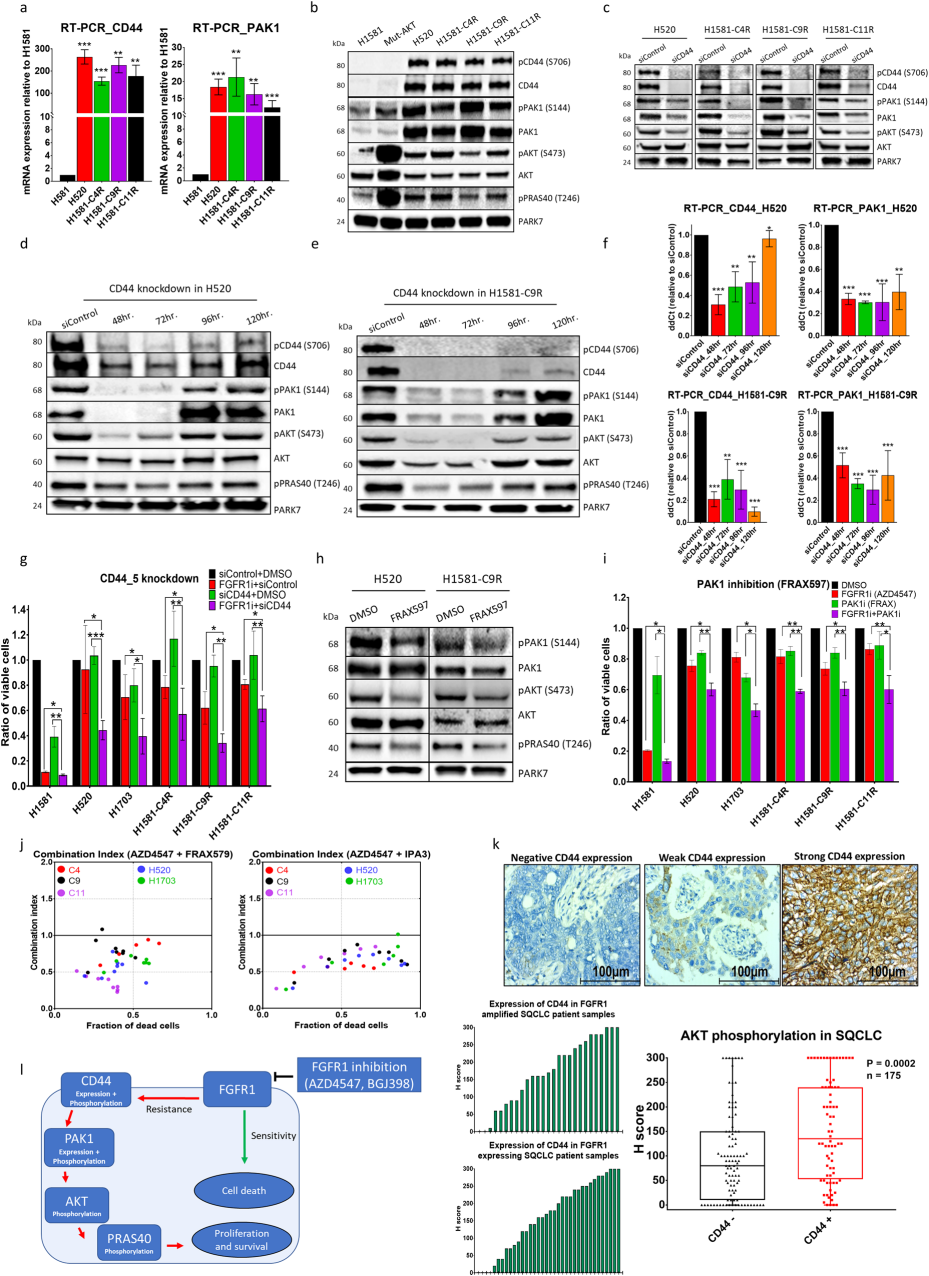
CD44/PAK1 overexpression and phosphorylation promotes FGFR1 inhibition resistance through AKT activation. **a** Real-time PCR to determine mRNA levels of CD44 and PAK1 in resistant cells. **b** Western blot analysis comparing levels of CD44, PAK1 and AKT among sensitive and resistant cells. **c** Western blot analysis shows the effect of CD44 knockdown among sensitive and resistant cells. **d**, **e** Effect of CD44 knockdown on PAK and AKT expression and activation in (**d**) H520 and (**e**) H1581-C9R cell lines. **f** Real-time PCR to determine mRNA levels of CD44 and PAK1 upon CD44 knockdown. **g** MTS assays show synergetic effect of combining CD44 knockdown (SI00299705) to FGFR1 inhibition in resistant cell lines. **h** Western blot analysis shows the effect of 3 h incubation of resistant cell lines with 1.2 µM o PAK1 inhibitor (FRAX597). **i** MTS assays show the synergetic effect of combining PAK1 inhibition (1.2 µM FRAX597) with FGFR1 inhibition in resistant cells. **j** Combination index plot showing the synergetic effect of combining two PAK1 inhibitors (IPA3 and FRAX597) with FGFR1 inhibitor (AZD4547). **k** Immunohistochemical staining of SQCLC patient samples with anti-CD44 antibody: correlation between CD44 expression and AKT phosphorylation within 175 lung cancer patients. **l** Proposed resistance axis to FGFR1 inhibition in lung cancer cells. CI plots were calculated according to the Chou–Talalay equation: CI < 1, synergetic effect; CI = 1, additive effect; CI > 1, antagonistic effect. Statistical analysis was performed with the chi-squared test: ns (*p* > 0.05), *(*p* < 0.05), **(*p* ≤ 0.01) and ***(*p* ≤ 0.001). Mean values are *p*lotted; error bars represent standard deviation.

Phosphoproteomic analysis of native (NCI-H520) and induced-resistant (H1581-9R and H1581-11R) lung-cancer cells compared with sensitive (NCI-H1581) and mutated AKT cell lines (mutant-AKT) revealed significant overphosphorylation of CD44 protein (by a factor of 30–50) at the site of serine-706 and PAK1 protein at site of serine-174 (Fig. 4h). Real-time PCR and western blotting showed that CD44 and PAK1 mRNA and proteins were strongly overexpressed and phosphorylated in the resistant cell lines compared with sensitive cells (Fig. 5a, b). To validate the correlation between CD44 expression and activation of PAK1 and AKT, we knocked down CD44 in the intrinsically resistant cell line (H520) and the three induced-resistant cell lines (H1581-C4R, C9R and C11R). CD44 knockdown led to a marked reduction of PAK1 expression, PAK1 phosphorylation and AKT phosphorylation (Fig. 5c–f). To examine the functional role of CD44 in FGFR1 inhibition resistance, we combined two different siRNAs targeting CD44 with FGFR1 inhibition in all resistant cells and observed a significant and strong reduction in cell viability within resistant cell lines (Fig. 5g and S8a). To test the influence of PAK1 activation on FGFR1 resistance, we inhibited PAK1 pharmacologically by using two different and selective inhibitors (IPA3 and FRAX597). PAK1 inhibition showed reduction of AKT activation within FGFR1-resistant cell lines and synergetic drop of resistant cells viability upon combination with FGFR1 inhibition (Figs. 5h–j and S8b, c). Finally, to validate the correlation between CD44 expression and AKT activation, we stained 175 SQCLC patient tissue samples with CD44 and pAKT antibodies and demonstrated a significant correlation between CD44 expression and AKT phosphorylation. Especially, strong expression of CD44 (H-score > 200), as had been observed in the cells that were resistant to FGFR1 inhibition, was strongly associated with the phosphorylation of AKT (Fig. 5k and Table 1). In conclusion, we propose that resistance to FGFR1 inhibition is induced through a signaling axis of that includes CD44, PAK1 and AKT (Fig. 5l).

## Discussion

Fibroblast growth factor receptor 1 (*FGFR1*) gene amplification is so far the most commonly detected and potentially targetable genetic alteration; it is found in ~22% of squamous cancers and ~7% of small-cell lung cancers^9,33^. Clinical trials have proven the efficacy of FGFR1 inhibitors in treating SQCLC patients. However, so far, resistance mechanisms have impeded favorable outcomes^15–17^.

In order to understand the complex signaling paths that lead to mechanisms of resistance to FGFR1 inhibition in lung cancer, we here performed for the first time a large-scale mass-spectrometric phosphoproteomic analysis of *FGFR1*-amplified lung cancer cells. We were able to demonstrate that activation of AKT induces resistance to FGFR1 inhibition. First of all, strong overphosphorylation of AKT, specifically at its activator phosphosites Ser473, Ser124 and Thr308, was found to be associated with resistance. In addition, strong phosphorylation of the proline-rich AKT substrate (PRAS40) as the main AKT downstream target was detected in resistant cells. PRAS40 is a regulator of the rapamycin complex 1 (mTORC1) pathway, and phosphorylation of PRAS40 inhibits its suppression of mTORC1 signaling, leading to its activation^34^. Secondly, while the SQCLC cell lines NCI-H1703 and NCI-H520 showed intrinsic resistance to FGFR1 inhibition and an inherent rise in AKT activation levels, 5.9–29.4% of ‘induced-resistant’ clones of the three former sensitive cell lines H1582, DMS114 and LK2 also showed elevated phosphorylation of AKT. Furthermore, mutationally induced AKT activation was able to convert FGFR1 inhibition sensitivity into resistance. Finally, combining inhibition of FGFR1 (AZD4547) and AKT (AZD5363, a selective ATP-competitive inhibitor) significantly increased the sensitivity of all resistant cells (intrinsic, induced and mutational resistance) to FGFR1 inhibition in a synergetic manner.

Targeting the PI3K/AKT/mTOR pathway is in general an interesting approach to tackling cancer progression, metastasis and treatment resistance, because of the vital role played by this pathway in these processes^34^. Nevertheless, it has been shown that inhibiting the AKT pathway as a single target is not very effective, owing to compensatory signaling loops^35,36^. However, combining AKT with other treatments has been shown to be effective in pancreatic cancer (gemcitabine), breast cancer (tipifarnib) and pulmonary adenocarcinoma (*EGFR*-TKI)^37–39^. As many different AKT kinase inhibitors have been developed and are currently being tested in different phases of clinical trials^35,40–42^, combining AKT and FGFR1 inhibition might be an option for patients who display resistance to FGFR1 inhibition with elevated AKT activation. Investigation of AKT phosphorylation at its activator site Ser473 in tissue samples from SQCLC patients confirmed high variability of the intrinsic AKT activation among those patients who harbored either *FGFR1* gene amplification or FGFR1 protein expression. Hence, intrinsic or induced activation of AKT could be the reason for the modest results found in clinical trials where FGFR1 inhibitors were tested.

A number of studies have been performed in the endeavor to understand the reason for, and mechanism of, FGFR1 resistance in FGFR1-amplified lung cancer patients^43–45^. However, to the best of our knowledge, the present study is the first to investigate the mechanism of FGFR1 resistance in a large-scale phosphoproteomic mass-spectrometry investigation, which included intrinsically resistant, induced-resistant and mutationally induced-resistant lung-cancer cells. Additionally, testing our FGFR1 resistance model against DNA- and RNA-directed lung cancer sequencing panels, as well as western blot analyses, allowed us to exclude previously described mechanisms of FGFR1 resistance such as AKT overexpression, KRAS mutation, PTEN abnormalities, FGFR1 gatekeeper mutations and MET overactivation^46,47^. On the other hand, phosphoproteomic analysis of the three different models of resistance to FGFR1 inhibition allowed investigation of pathways upstream and downstream of AKT and led to the identification of CD44 and PAK1 overexpression and activation as a common resistance mechanism in both intrinsic and induced resistance to inhibition of FGFR1.

CD44 is an adhesion glycoprotein that plays important roles in breast, colorectal, thyroid and lung cancer progression and metastasis^22,48,49^. PAK1 is a protein kinase that is involved in different signaling pathways in healthy cells (e.g., proliferation, cell adhesion and migration), and its overactivation emphasizes chemoresistance in lung cancer^50,51^. Moreover, the correlation between CD44 and PAK1 activation was reported before in colorectal cancer, where PAK1 activation correlated to CD44 expression levels and promoted chemoresistance^52^. Furthermore, our staining of CD44 in SQCLC patients’ tissue samples showed a significant correlation between CD44 expression and AKT activation in human tissue, which constitutes further evidence that CD44 plays a part in activating AKT and hence inducing resistance to FGFR1 inhibition in these patients.

In conclusion, we used a phosphoproteomic approach to investigate diverse resistance mechanisms to FGFR1 inhibition in lung-cancer cells. In addition to providing a large data library of resistance-associated phosphorylation patterns, our results lead us to propose a common resistance pathway that includes the activation of CD44, PAK1 and AKT. Examination of CD44/PAK1/AKT activation could help to predict response to FGFR1 inhibition, and combination with AKT or PAK1 inhibitors might pave the way toward an effective therapy for FGFR1-dependent lung-cancer patients in cases of resistance to treatment.

## Methods

### Tissue samples

Approval for using patient tissue samples from surgical resections at the Department of Thoracic Surgery of the University Medical Center Goettingen was obtained from the institutional Ethics Committee of University Medical Center of Goettingen (#1-2-08). All patients gave informed written consent to take part in the study. All procedures were designed and performed in compliance with the Declaration of Helsinki and all institutional, state and federal guidelines.

### Immunohistochemistry (IHC)

IHC and cell blocks were prepared as described earlier^53,54^. Staining was performed on 2 µm-thick paraffin sections, which were incubated with Target Retrieval Solution (EnVision Flex, Dako, Carpinteria, California, USA) at pH 9 and then with primary antibodies (anti-FGFR1 (Abcam, Berlin, Germany), dilution 1:5000, anti-pAKT (Abcam), 1:100, or anti-CD44 (Sigma–Aldrich, Taufkirchen, Germany), 1:1000, at room temperature for 20 min. Secondary antibody was visualized by using DAB substrate, and contrasting was achieved by hematoxylin staining.

### Fluorescence in situ hybridization (FISH)

FISH was conducted and assessed according to published protocols^55^. Briefly, sections 3–4 µM thick were cut from blocks and mounted on slides. Deparaffinization, protease treatment and washing were carried out with a VP2000 processor system. ZytoLight SPEC (*FGFR1*/CEN8, ZYTOVISION, Z-2072-200) was used to hybridize cells overnight at 37 °C; staining was performed with DAPI.

### Cell culture

DMS114 (NCI, Bethesda, MD, USA), NCI-H1581 and NCI-H1703 (AddexBio, San Diego, CA, USA), LK-2 (JCRB, Tokyo, Japan), NCI-H520 (ATCC, Wesel, Germany) and HCC-15 (DSMZ, Braunschweig, Germany) lung-cancer cell lines were cultured in RPMI-1640 media (Gibco, Grand Island, NY, USA) with 10% fetal bovine serum plus 1% glutamine plus 1% penicillin/streptomycin (Gibco) and incubated in 5% CO_2_ at 37 °C. Trypsin–EDTA (0.05%, Gibco) was used for detachment.

### Inhibitors and viability assays

AZD4547, AZD5363, BGJ398, FRAX597 and IPA3 were purchased from Selleckchem, USA. For the MTS cell-viability assay, cell lines were seeded at appropriate density in 96-well plates for 24 h and then treated with either DMSO or inhibitor for 96 h in 5% CO_2_ at 37 °C. Viability was measured by adding 20 µL per well of cell titer Aqueous One solution (Promega, Walldorf, Germany) for 2.5 h in 5% CO_2_ at 37 °C. Absorbance was detected at 660 nm wavelength with a TECAN 200 M pro (TECAN, Zuerich, Switzerland). For counting of viable cells, cells were treated with either DMSO or inhibitor for the appropriate time, whereafter they were detached and counted by using a Guava Muse cell analyzer (Luminex, USA); counting was based on cell size and nucleation status.

### Western blotting

Western blotting analyses were performed as described before^9^. Briefly, cells were harvested and lysed for 20 min on ice in 10 mM Tris HCl, 1% NP-40, Complete EDTA (Roche, Basel, Switzerland) and 0.1% sodium orthovanadate (Sigma–Aldrich) at pH 8. Protein concentration was assayed with the DC protein assay (Bio-Rad, Feldkirchen, Germany). 10–25 µg of denatured proteins were loaded onto mini-protean precast gels (4–20%; Bio-Rad). Protein was blotted onto nitrocellulose membranes through Trans-Blot Turbo (Bio-Rad), blocked in 5% milk and incubated with primary antibodies overnight at 4 °C (Cell signaling: CD44#3570 (1:1000), Phospho-PRAS40 (Thr246)#13175 (1:1000), Phospho-FGFReceptor1(Tyr653/654)#52928 (1:1000), FGF Receptor1#9740 (1:1000), Phospho-AKT(Ser473)#4060 (1:1000), AKT#9272 (1:1000), Phospho-FoxO1 (Thr24)/FoxO3a(Thr32)#9464 (1:1000), Phospho-PAK1 (S144)#2606 (1:1000), PAK1 #2602 (1:1000), Phospho-MET (Y1234/1235)#3077 (1:1000), MET# 4560 (1:1000), Phospho-MAPK(Thr202/Tyr204)#9101 (1:1000), MAPK#9102 (1:1000), Phospho-MEK1/2(Ser217/221)#9121 (1:1000), MEK1/2#9122 (1:1000), Abcam: Anti-PARK7/ab18257 (1:1000), Merck: Anti-phospho-CD44 (pSer706)SAB4504135) (1:20000). Membranes were washed 3× for 30 min with TBST buffer followed by incubation with secondary antibodies (Agilent, Santa Clara, CA, USA). Reblot Plus strong (Merck, Darmstadt, Germany) was used in cases were sequential staining was needed. Bands were visualized by using Western Plus-ECL (PerkinElmer, Waltham, MA, USA) on Fusion Fx and peQlab camera (VILBER, Collegien, France). PARK7 was used as a housekeeping protein owing to its highly stable expression in diverse human tissues and cell lines^56^.

### Overexpression vectors

DH5 alpha bacteria were transformed with either pcDNA3-Myr-AKT1 (plasmid #9008) or an empty pcDNA3 vector (plasmid #1079) from Addgene, USA. Bacteria were then amplified overnight in LB medium (Carl Roth GmbH, Germany). Plasmids were extracted by using the Maxiprep kit (Qiagen, Nederland) following its standard protocol. Cells were transfected in 6-well plates with 2.5 µg of either vector after complexing with 7 µL Lipofectamine3000 (Invitrogen, Waltham, MA, USA) in 250 µL serum-free medium. Transfection lasted for 10 h, and the medium was then replaced with fresh medium containing the antibiotic geneticin (Invivogen, San Diego, CA, USA; 100–1000 µg/mL) and cells were kept under selection medium for the duration of experiments. All blots on the same membrane were derived from the same experiment and were processed in parallel.

### Apoptosis assay

Cells were seeded on Day 1 in 6-well plates and treated with the corresponding concentration of DMSO or AKT inhibitor on day 2 Day 2 with an incubation time of 24 h. Cells were washed with cold PBS, re-suspended in binding buffer (422201, Biolegend, USA) and 5 µL of Annexin V stain was added to 100 µL of suspended cells with gentle mixing. Propidium iodide (10 µL) was added to the cells, which were incubated for 15 min in the dark. Cells were diluted with 400 µL binding buffer and analyzed on a FACS analyzer.

### Knockdown using siRNA

All siRNAs were purchased from GeneGlobe Qiagen (Venlo, Netherlands). Verified siRNAs targeting FGFR1 and CD44 were used (Hs_CD44_5 FlexiTube, Geneglobe ID; SI00299705, Hs_CD44_9 FlexiTube, Geneglobe ID; SI03062661, Hs_FGFR1_6 FlexiTube, Geneglobe ID; SI02224677 and Hs_FGFR1_7 FlexiTube Geneglobe ID; SI02224684). Cell lines were transfected with Hiperfect as a transfection reagent (Qiagen) according to the manufacturer’s protocol. For control cells, a scrambled control siRNA was used (Allstars negative control siRNA, Qiagen). To test transfection efficiency of different cell lines using Hiperfect, a control siRNA conjugated to a fluorescent dye (Alexa Fluor 488) was used. Transfection efficiency was measured by flow cytometry (BD, Franklin Lakes, NJ, USA). Briefly, siRNAs were diluted at the appropriate concentration, complexed with Hiperfect for 10 min at room temperature and then added dropwise to the cells. Viability of cells was measured with propidium iodide.

### DNA extraction, Sanger sequencing and real-time PCR

DNA was extracted by using QIAamp DNA Mini Kit (Qiagen). For sequencing, the BigDye^TM^ Terminator v3.1 sequencing kit (ThermoFischer, Waltham, MA, USA) was used, following the manufacturer’s protocol. Briefly, target sequence was amplified by PCR and then purified with the ExoSAP clean-up kit (ThermoFischer). The BigDye XTerminator™ purification kit was used for cleaning ahead of sequencing with a 3500DX Genetic Analyzer (ThermoFisher) and analyzing results with the Geneious prime 2019 software. Real-time PCR was performed using qPCRBIO SyGreen Mix kit from PCRBIOsystems. Following primers were used: CD44: Fwd: TCCAACACCTCCCAGTATGACA, Rev: GGCAGGTCTGTGACTGATGTACA and PAK1: Fwd: TTACGGGAATGCCAGAGCAG, Rev: CAGCCTGCGGGTTTTTCTTC.

### Archer fusionPLEX CTL sequencing panel

Samples were prepared as published earlier^57^. DNA (200–500 ng) was extracted, sheared to sizes of ~300 base pairs with a S2-Covaris Focused-ultrasonicator and paired to the NEBNext Ultra II DNA Library Kit following the manufacturer’s protocol. An average of 17.1 million sequences per sample were measured by using NextSeq500 (Illumnia, San Diego, CA, USA) and bcl2fastq software (version 2.17.1.14), and mapped to HG19 (human reference genome).

### SILAC metabolic labeling

SILAC labeling of cancer cell lines was performed as described earlier^53^. Briefly, cells were cultured in SILAC RPMI 1640 medium (Gibco, USA) devoid of arginine and lysine and containing 10% dialyzed FCS (Gibco, USA), 1% PenStrep (Gibco, USA), 1% glutamine (Gibco, USA) and 1% sodium pyruvate (Sigma–Aldrich, Germany). For SILAC, the medium was supplemented with medium-heavy (^13^C_6_ arginine and D_4_ lysine, Cambridge Isotope Laboratories, Inc., Tewksbury, MA, USA), heavy (^13^C_6_^15^N_4_ arginine and ^13^C_6_^15^N_2_ lysine, Cambridge Isotope Laboratories, Inc) or light (unlabeled) amino acids. All cells were labeled for at least 10 cell divisions and were then treated with DMSO or with AZD4547 for 3 h before lysis.

### Cell lysis and sample preparation for phospho-profiling

Cells were lysed with global phosphoproteome (GPome) or tyrosine enrichment (pYome) lysis buffers. The buffer for GPome analyses contained 50 mM Tris-HCl (pH 5.5), 1 mM EDTA, 1% NP-40, protease inhibitor cocktail (Roche), 5 mM NaF, 10 mM NEM and LC/MS-grade water. The buffer for pYome analyses was prepared according to the manufacturer’s protocol (P-Tyr-1000 kit, Cell Signaling Technology, Danvers, MA, USA). Cells were scraped into PBS (ice-cold) and incubated with the appropriate lysis buffer for 20 min on ice (GPome) or at room temperature (pYome).

For GPome analyses the cell suspensions were sonicated for 3× 10 s and centrifuged for 15 min at 4 °C and 20,000 × *g*. For each SILAC condition, an aliquot corresponding to 1 mg protein was taken from the supernatant; aliquots were mixed and precipitated with acetone at –20 °C overnight. The dried proteins were suspended in urea buffer (8 M urea, 20 mM HEPES, pH 8.0, phosphatase inhibitors), followed by reduction with DTT and alkylation with iodoacetamide. For protein digestion, the samples were incubated with Lys-C (Wako, Kanto, Japan) in an enzyme-to-substrate ratio of 1:100 (w/w) at 37 °C for 2 h and, after dilution to 2 M urea, with trypsin (Promega) at 37 °C overnight in an enzyme-to-substrate ratio of 1:100 (w/w). The peptide samples were purified by using Sep-Pak C18 classic cartridges (360 mg, Waters) and dried by vacuum centrifugation.

For pYome analyses sample preparation was carried out according to the manufacturer of the deployed enrichment kit (P-Tyr-1000 kit, Cell Signaling Technology). Briefly, the cell suspensions were sonicated and supernatants from SILAC conditions were mixed in amounts corresponding to equal protein quantities, followed by reduction with DTT and alkylation with iodoacetamide. After dilution to 2 M urea with 20 mM HEPES pH 8.0, the protein samples were digested with trypsin (Promega) in an enzyme-to-substrate ratio of 1:100 (w/w) at 37 °C overnight. The peptide samples were purified by using Sep-Pak C18 classic cartridges and lyophilized.

### Phosphopeptide enrichment

For GPome analyses phosphopeptides were enriched from the dried peptide samples by using titanium dioxide affinity chromatography by the spin-tip method (Pierce High-Select TiO_2_ phosphopeptide enrichment kit, Thermo Fisher Scientific) according to the manufacturer’s instructions. Briefly, the peptides were suspended in 150 µl of the kit buffer and subjected to phosphopeptide enrichment and purification. After eluting the phosphopeptides from the spin columns, samples were immediately dried by vacuum centrifugation and subjected to high-pH RPLC pre-fractionation (Pierce High pH RPLC fractionation kit, Thermo Fisher Scientific).

For pYome analyses, the lyophilized peptide samples were suspended in 1.4 ml of IAP buffer, and tyrosine-phosphorylated peptides were immunoprecipitated with the P-Tyr-1000 antibody kit (Cell Signaling Technology) according to the manufacturer’s instructions. Enriched phosphopeptides were eluted under acidic conditions, purified by using C18 RPLC microtips and dried by vacuum centrifugation.

### Mass spectrometry and data analysis

Mass-spectrometric analyses were conducted basically according to published procedures^58,59^. Phosphopeptide samples were analyzed on a Q Exactive HF orbitrap mass spectrometer (Thermo Fisher Scientific) coupled to an Ultimate 3000 RSLCnano HPLC system (Dionex/Thermo Fisher Scientific) through a nano-ESI interface (Nanospray flex, Thermo Fisher Scientific). First, the peptides were trapped on a 100 µm × 5 cm pre-column (ReproSil-Pur 120 C18-AQ, 5 µm; Dr. Maisch GmbH, Ammerbuch, Germany) and then separated on a 30.5 × 0.075 mm analytical column (ReproSil-Pur 120 C18-AQ, 1.9 μm; Dr. Maisch GmbH) at a flow rate of 300 nl/min with a 90-min (GPome) or 120-min (pYome) linear gradient of 2–40% solvent B (80% v/v ACN, 0.1% FA) over solvent A (0.1% FA). Peptides eluted from the column were ionized and analyzed in data-dependent acquisition mode using a TopN MS/MS method with a survey scan resolution setting of 120,000 FWHM and an MS/MS resolution setting of 30,000 FWHM at 200 *m/z*, respectively. The 15 (for pYome measurements: 20) most abundant ions within the *m/z* range 350–1600 and having charge states of 2–6 were selected for HCD with an NCE setting of 28% and an isolation width of 1.6 *m/z*. AGC target values and maximum ion injection times for MS and MS/MS were set to 1 × 10^6^ in 50 ms (for pYome: 40 ms) and 1 × 10^5^ in 110 ms (128 ms), respectively. Selected precursor mass-to-charge ratio values were dynamically excluded from fragmentation for 30 s (20 s).

Raw data files were analyzed with the MaxQuant software (version 1.6.5.0, Max Planck Institute for Biochemistry). Mass spectra were searched, with the integrated Andromeda search engine, against the UniProtKB human reference protein database (date: February 2019) supplemented with 245 common contaminants. Trypsin was set as enzyme for protein digestion, and the precursor and fragment-ion mass tolerances were set to 4.5 and 20 ppm, respectively. Oxidation of methionine, protein *N*-terminal acetylation and phosphorylation of serine, threonine and tyrosine were allowed as variable modifications. Carbamidomethylation of cysteine was set as a fixed modification. The minimum peptide length was set to seven amino acids, allowing two missed cleavages. On both the peptide and protein level the maximum false discovery rate was set to 1% on the basis of a decoy database search. SILAC multiplicity was set to triple labeling (Lys+0/Arg+0, Lys+4/Arg+6, Lys+8/Arg+10) and at least two ratio counts were required for peptide quantification. Furthermore, the “re-quantify” option was enabled^60^. The mass spectrometry proteomics data have been deposited with the ProteomeXchange Consortium, through the PRIDE partner repository, with the dataset identifier PXD025389^61^.

For downstream data analysis the Perseus software (version 1.6.10.43) was used. Phosphosites identified were filtered for potential contaminants, hits to the decoy database and localization probability above 0.75. Technical replicates were grouped by their average, and biological replicates were used to calculate the statistical significance. Data were compensated for missing values and z-scored to generate heatmaps according to analysis of variance (ANOVA).

### Statistical analysis

Pearson’s coefficient and the chi-squared test were used to investigate the correlation between AKT phosphorylation, patient characteristics and CD44 expression. *p* values were calculated by using the chi-squared, Mantel–Cox and Student’s *t* tests. Significant *p* values were defined as those below 0.05. The effect of combining two or more inhibitors was calculated by the Chou-Talalay method (CompuSyn, version 1.0), with CI < 1 implying synergy, CI = 1 addition and CI > 1 antagonism^21^.

### Reporting summary

Further information on research design is available in the Nature Research Reporting Summary linked to this article.

## Supplementary information

Supplementary_Information

Supplementary_table_1

Supplementary_table_2

Supplementary_table_3

REPORTING SUMMARY

## Data Availability

Raw reads of RNA CTL sequencing and Hot Spot DNA sequencing have been uploaded to the European Nucleotide Archive (ENA) data repository with accession number: PRJEB52872. Raw and processed mass-spectrometry data have been submitted to the PRIDE proteomics data repository (http://www.ebi.ac.uk/pride/archive/), Project accession: PXD025389. Further raw data of the current study are available on request from the corresponding author.
