# Supplementary material for: Activation of CD44/PAK1/AKT signaling promotes resistance to FGFR1 inhibition in squamous-cell lung cancer
Source: NPJ Precis Oncol. 2022 Jul 19;6:52. doi: 10.1038/s41698-022-00296-2 (PMC9296622; doi:10.1038/s41698-022-00296-2)

Supplementary Figure 1

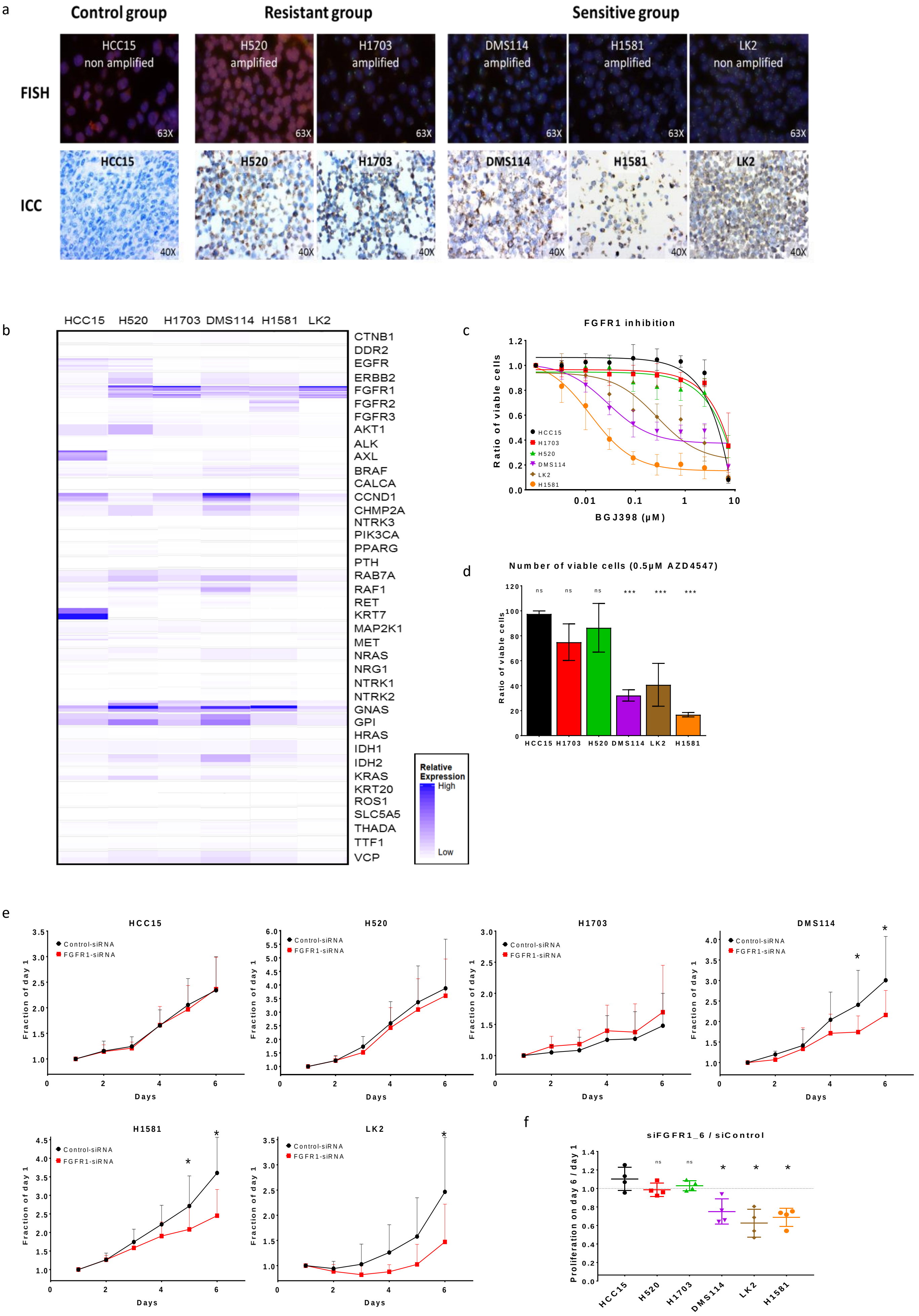

Supplementary Figure 1: (A) Fluorescence *in situ* hybridization analysis comparing numbers of green (FGFR1) and orange (CEN8) signals in nuclei of control lung cancer cell line (HCC15), resistant cell lines (NCI-H520 and NCI-H1703) and sensitive cell lines (DMS114, H1581 and LK2) to FGFR1 inhibition (top) and immunocytochemistry staining of FGFR1 expression in these cell lines (bottom). **(B)** RNA sequencing comparing control, resistant and sensitive cell lines using Archer thyroid and lung cancer directed panel. **(C)** Cell viability assay of the named six cell lines after treatment with the FGFR1 inhibitor BGJ398 measured after 96 hours. **(D)** Viable cell numbers counted after treatment of the named six cell lines with the FGFR1 inhibitor AZD4547 at concentration of 0.5 µM for 96 hours. **(E)** Proliferation of the cell lines was measured for 6 days after treatment with siRNA targeting FGFR1 and compared with cells treated with control scrambled siRNA. **(F)** FGFR1 knockdown using a validated siRNA sequence (SI02224677). Statistical analysis was performed with the Chi-square test: ns (p>0.05), * (p<0.05), ** (p≤0.01) and *** (p≤0.001). Mean values were plotted and error bars represent standard deviation.

Supplementary Figure 2

c

Inhibitor (μM)	1	2	3	4	5	6	7	8	9
FGFR1 (AZD4547)	DMSO	0.003	0.01	0.03	0.1	0.27	0.8	2.4	7.3
AKT (AZD5363)	DMSO	0.50	1	5	10	15	20	25	30

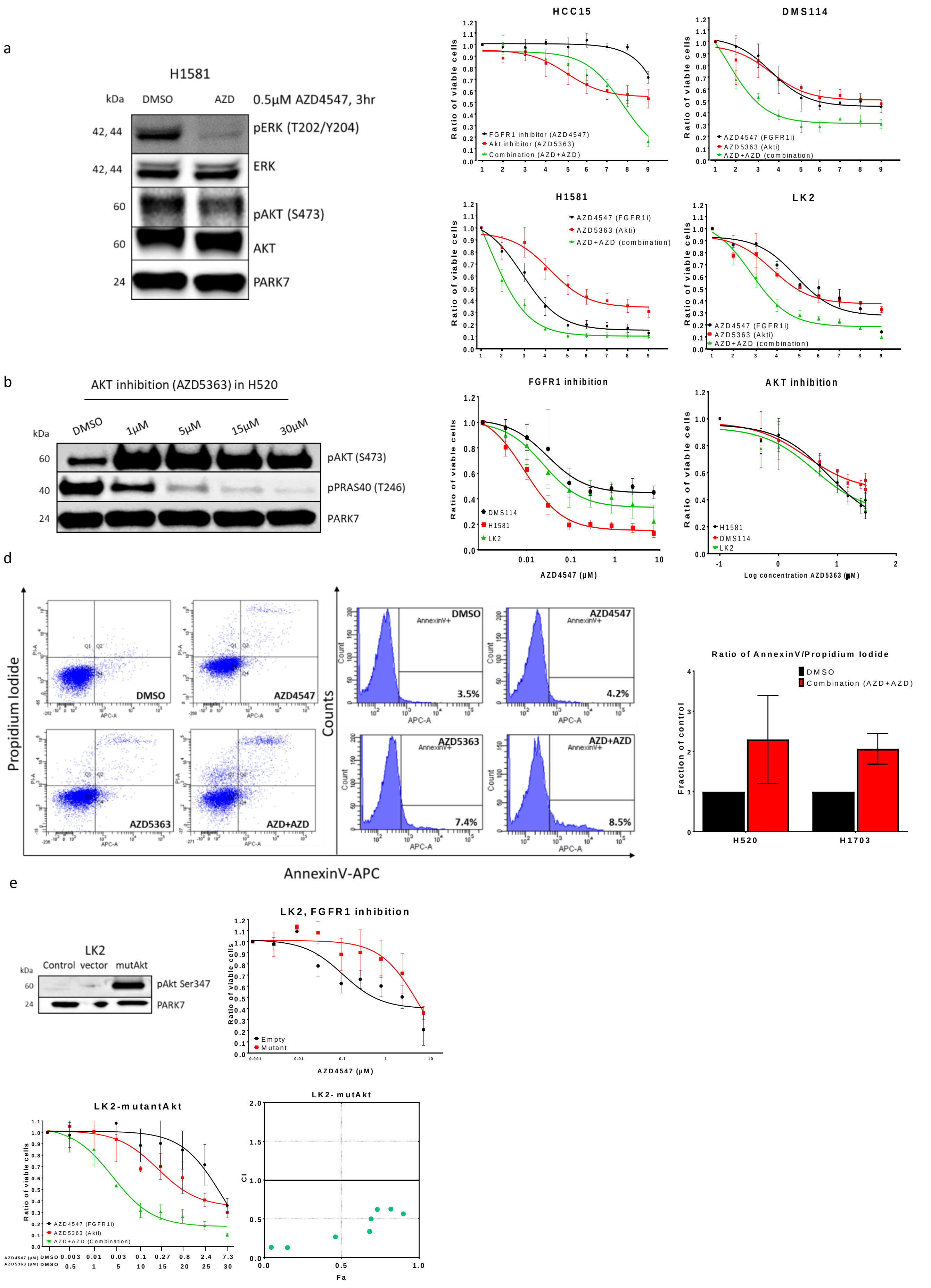

Supplementary Figure 2: (A) Western blot analysis showing effect of FGFR1 inhibition on AKT activation. **(B)** The inhibition effect of a gradient of AKT inhibitor concentrations. **(C)** Cell viability combination assays showing the effect of combining FGFR1 inhibition (AZD4547) with AKT inhibition (AZD5363) in control cell lines and cell lines sensitive to FGFR1 inhibition. **(D)** Images show the effect of combination inhibition of FGFR1 (AZD4547) and AKT (AZD5363) on inducing cellular apoptosis by measuring levels of ANNXINV using FACS analyzer. **(E)** Western blot and Cell viability assays showing the effect of either single or double treatment with AZD454 (FGFR1 inhibitor) and AZD5363 (AKT inhibitor) in the control LK2 cell line transfected with an empty backbone vector or a vector with constitutive active AKT gene. The nature of the interaction between the two inhibitors as indicated by a combination index plot (Chou-Talalay: (CI < 1, synergetic effect; CI = 1, additive effect; CI > 1, antagonistic effect). Statistical analysis was performed with the chi-squared test: ns (p>0.05), * (p<0.05), ** (p≤0.01) and *** (p≤0.001). Mean values are plotted and error bars represent standard deviation.

Supplementary Figure 3

a

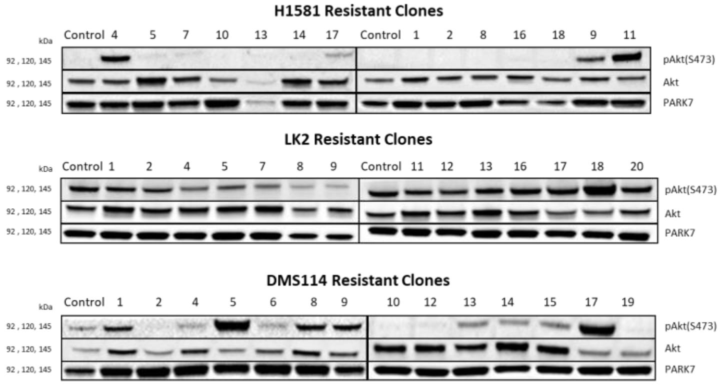

b

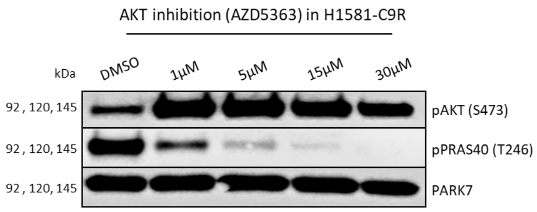

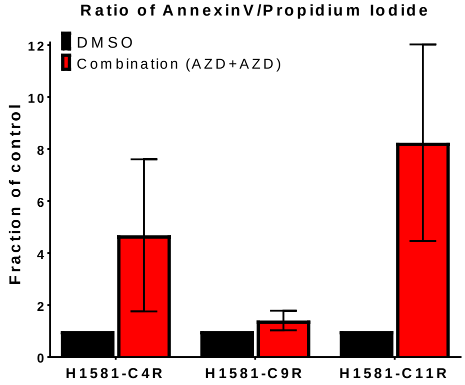

D

Gene	(exons)	Parental	Clone 4R	Clone 9R	Clone 11R
Akt1	-3	Wild type	Wild type	Wild type	Wild type
ALK	22-25	Wild type	Wild type	Wild type	Wild type
BRAF	11 & 15	Wild type	Wild type	Wild type	Wild type
CTNNB1	3	Wild type	Wild type	Wild type	Wild type
EGFR	18-21	Wild type	Wild type	Wild type	Wild type
ERRB2	8, 12, 14, 17-21, 24&26	Wild type	Wild type	Wild type	Wild type
FGFR1	4-7, 10 & 12-15	Wild type	Wild type	Wild type	Wild type
FGFR2	7, 8, 10, 11 & 13-15	Wild type	Wild type	Wild type	Wild type
FGFR3	3, 6, 9, 12, 13, 15 &16	Wild type	Wild type	Wild type	Wild type
FGFR4	3, 6, 9, 12, 13, 15 & 16	Wild type	Wild type	Wild type	Wild type
HRAS	2-4	Wild type	Wild type	Wild type	Wild type
IDH1	4	Wild type	Wild type	Wild type	Wild type
IDH2	4	Wild type	Wild type	Wild type	Wild type
KRAS	2-4	Wild type	Wild type	Wild type	Wild type
MAP2K1	2	Wild type	Wild type	Wild type	Wild type
MET	14, 16-19 & intron 13	Wild type	Wild type	Wild type	Wild type
NFE2L2	2	Wild type	Wild type	Wild type	Wild type
NRAS	2-4	Wild type	Wild type	Wild type	Wild type
PIK3CA	10 & 21	Wild type	Wild type	Wild type	Wild type
PTEN	1-8	Wild type	Wild type	Wild type	Wild type
RET	1-20	Wild type	Wild type	Wild type	Wild type
ROS1	34-41	Wild type	Wild type	Wild type	Wild type
TP53	4-11	Mutant	Mutant	Mutant	Mutant
SMAD4	3 & 9-12	Wild type	Wild type	Wild type	Wild type

E

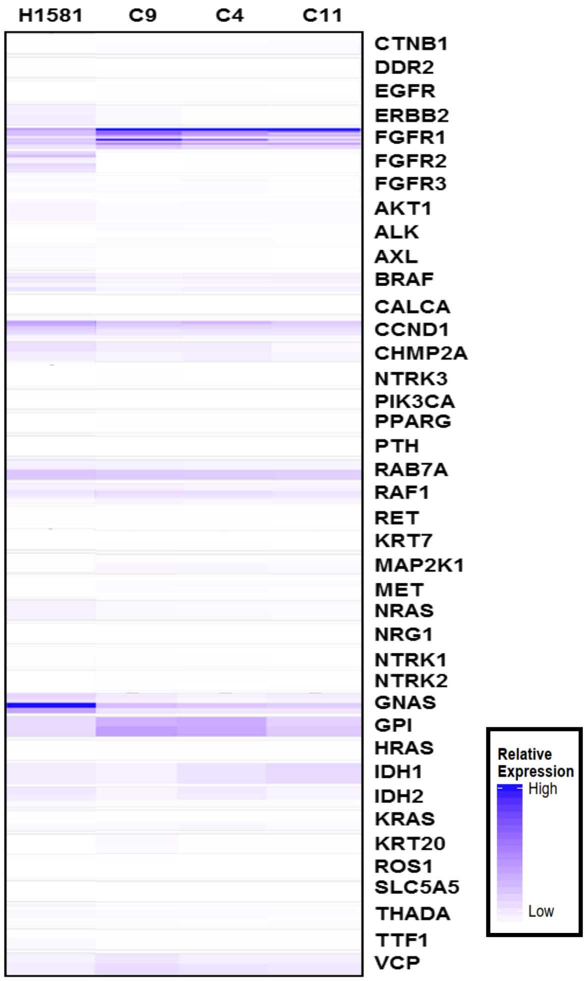

Supplementary Figure 3: (A) Western blot analysis showing the expression of FGFR1, phosphorylated AKT, total AKT, phosphorylated ERK and total ERK among different single clones with induced FGFR1 inhibition resistance. **(B)** Western blot analysis showing the effect of grading concentration of AKT inhibition in resistant cell lines. **(C)** Effect of double treatment (FGFR1 inhibitor, AZD4547 and AKT inhibitor; AZD5363) on cellular apoptosis in FGFR1 resistant cell lines. **(D)** DNA targeted sequencing panel against main gene alterations in lung cancer comparing the sensitive parental control cell line H1581 with the three single induced resistant clones H1581-C4R, H1581-C9R and H1581-C11R. **(E)** RNA sequencing using the Archer thyroid- and lung-cancer-directed panel comparing RNA expression among the sensitive parental control cell line and its three resistant single clones. Statistical analysis was performed with the Chi-square test: ns (p>0.05), * (p<0.05), ** (p≤0.01) and *** (p≤0.001). Mean values were plotted and error bars represent standard deviation.

Supplementary Figure 4

a

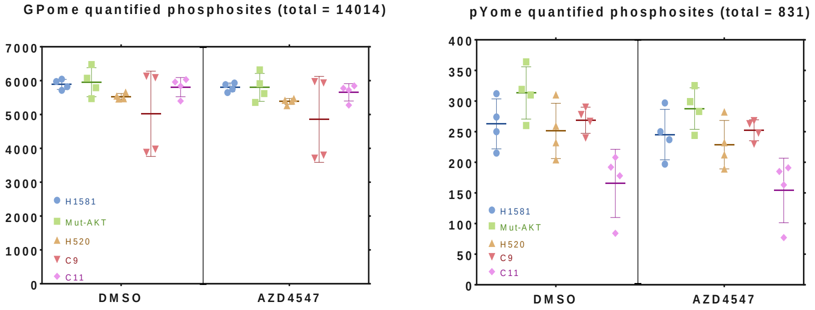

b

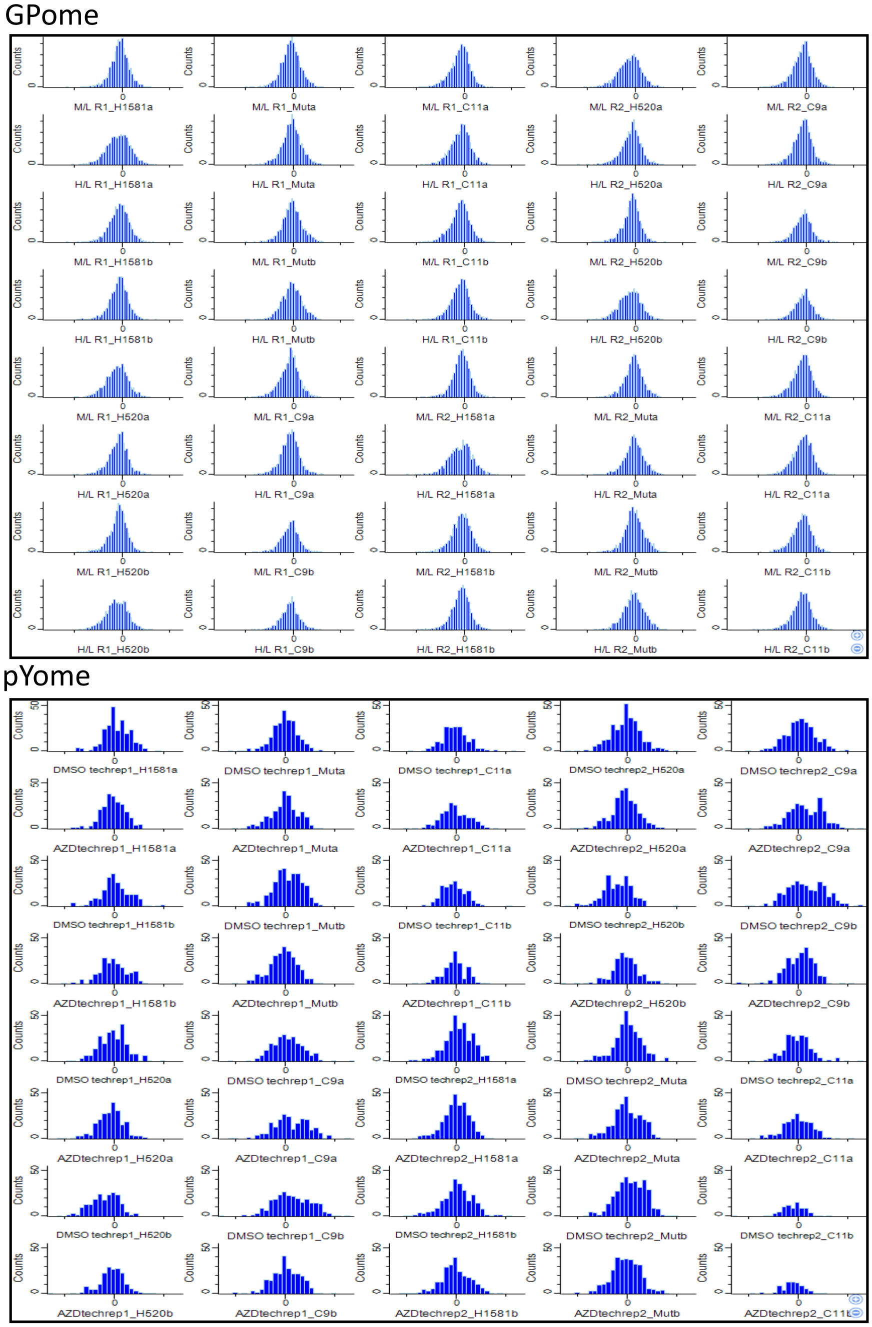

Supplementary Figure 4: (A) Numeric Venn diagram showing number of quantified phosphosites among the five cell lines used in phospho-LC-MS/MS analysis after GPome and pYome enrichment using two technical and two biological replicates for each sample. **(B)** Histograms showing normal distribution of SILAC ratios among the five cell lines used in LC-MS/MS analysis; **a** and **b** indicate biological replicates while R1 and R2 indicate technical replicates for each sample. Mean values are plotted; error bars represent standard deviation.

Supplementary Figure 5

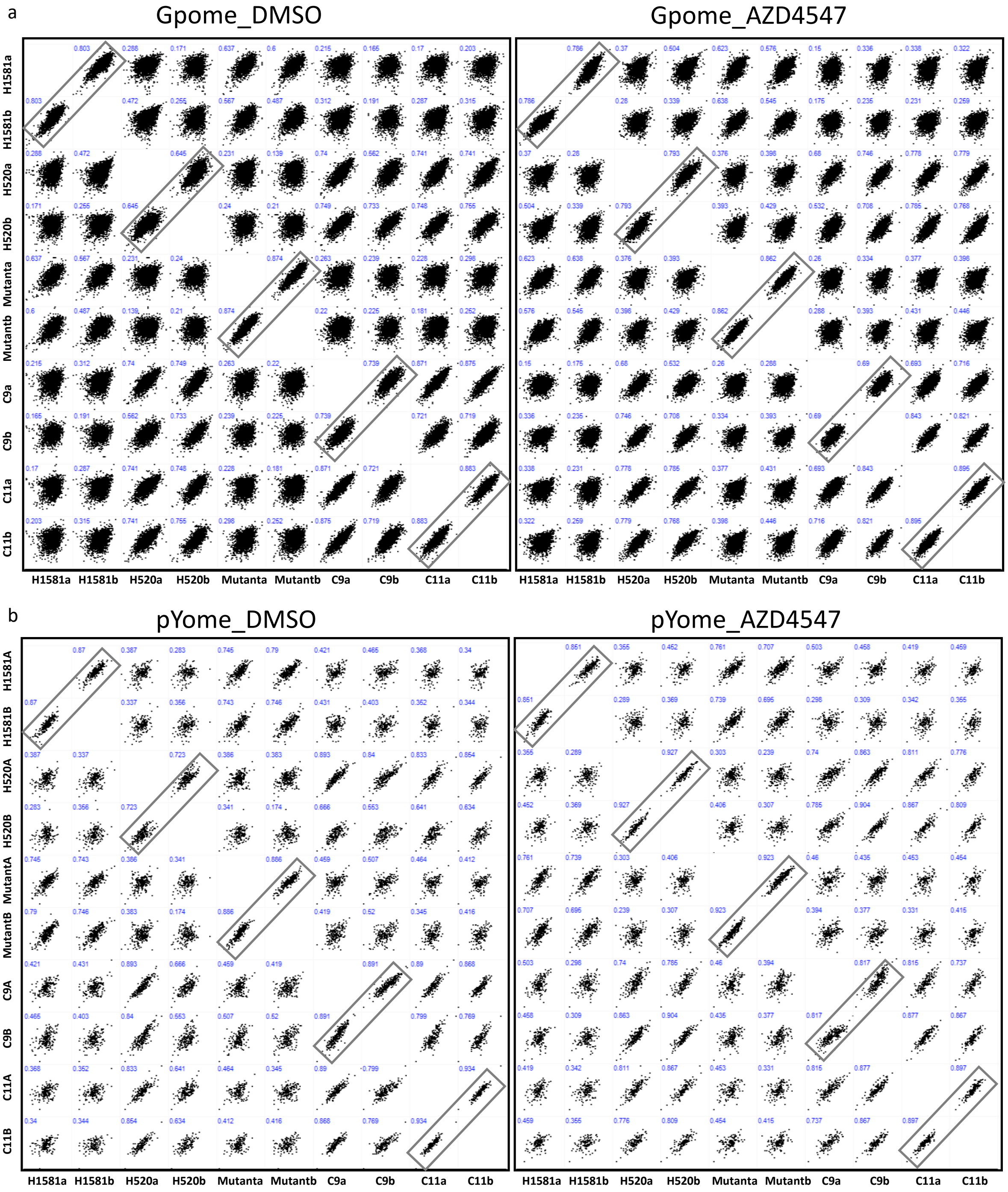

Supplementary Figure 5: Scatter plots showing similarity between biological replicates for each sample used in the phospho LC-MS/MS analysis through Pearson correlation; **a** and **b** indicate first and second biological replicates, respectively, in the GPome (A) and pYome (B) group.

Supplementary Figure 6

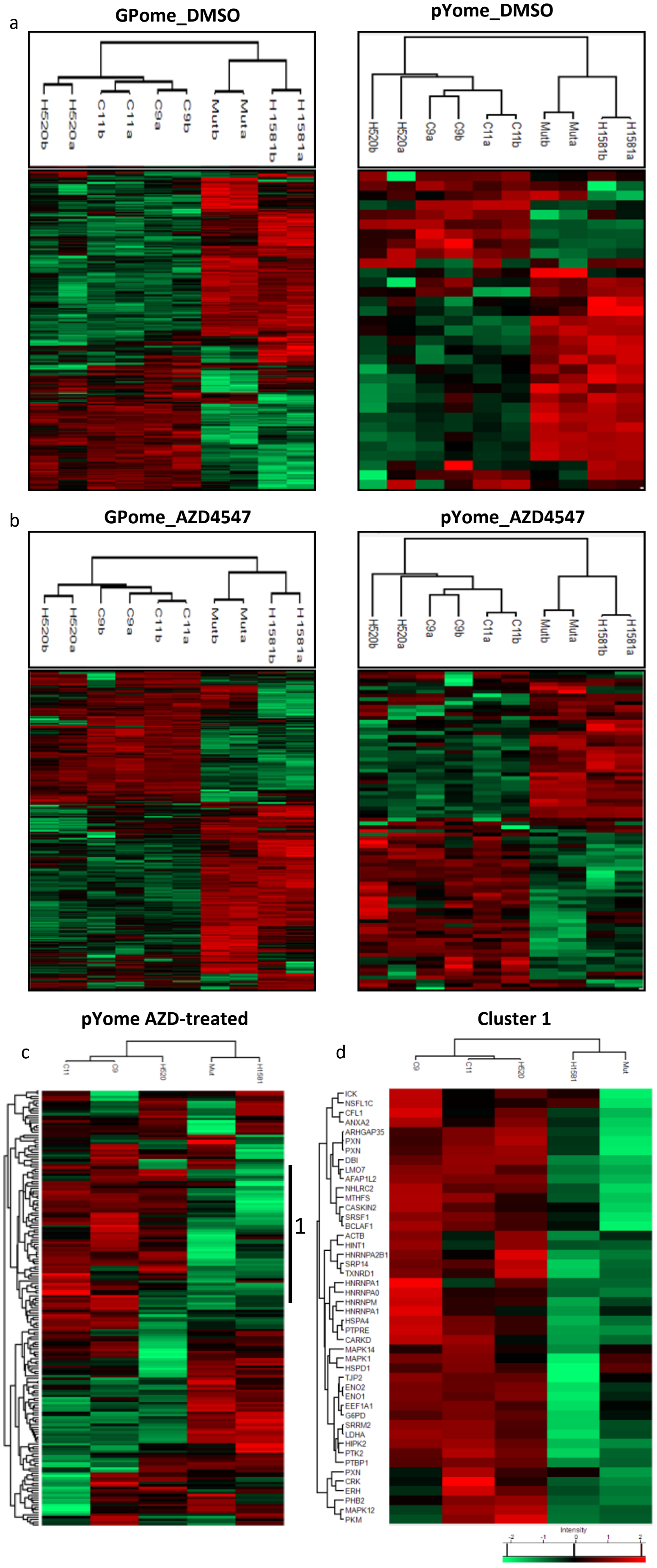

Supplementary Figure 6: Heatmaps showing significantly up- and down-regulated phosphosites among each of the biological replicates of the five cell lines. **(A, B)** Biological replicates among DMSO-treated (A) and AZD4547-treated (B) cells. **(C, D)** Heatmaps showing significantly up- and down-regulated phosphorylation in: the native-resistant cell line NCI-H520; the induced-resistant H1581 with resistance induced by AKT mutational overexpression; two induced-resistant single clones H1581-C9R and H1581-C11R; and the sensitive parental control H1581 cell after treatment with the FGFR1-inhibitor AZD4547 and pYome enrichment (C) and expansion of the indicated cluster 1 (D).

Supplementary Figure 7

a GPome

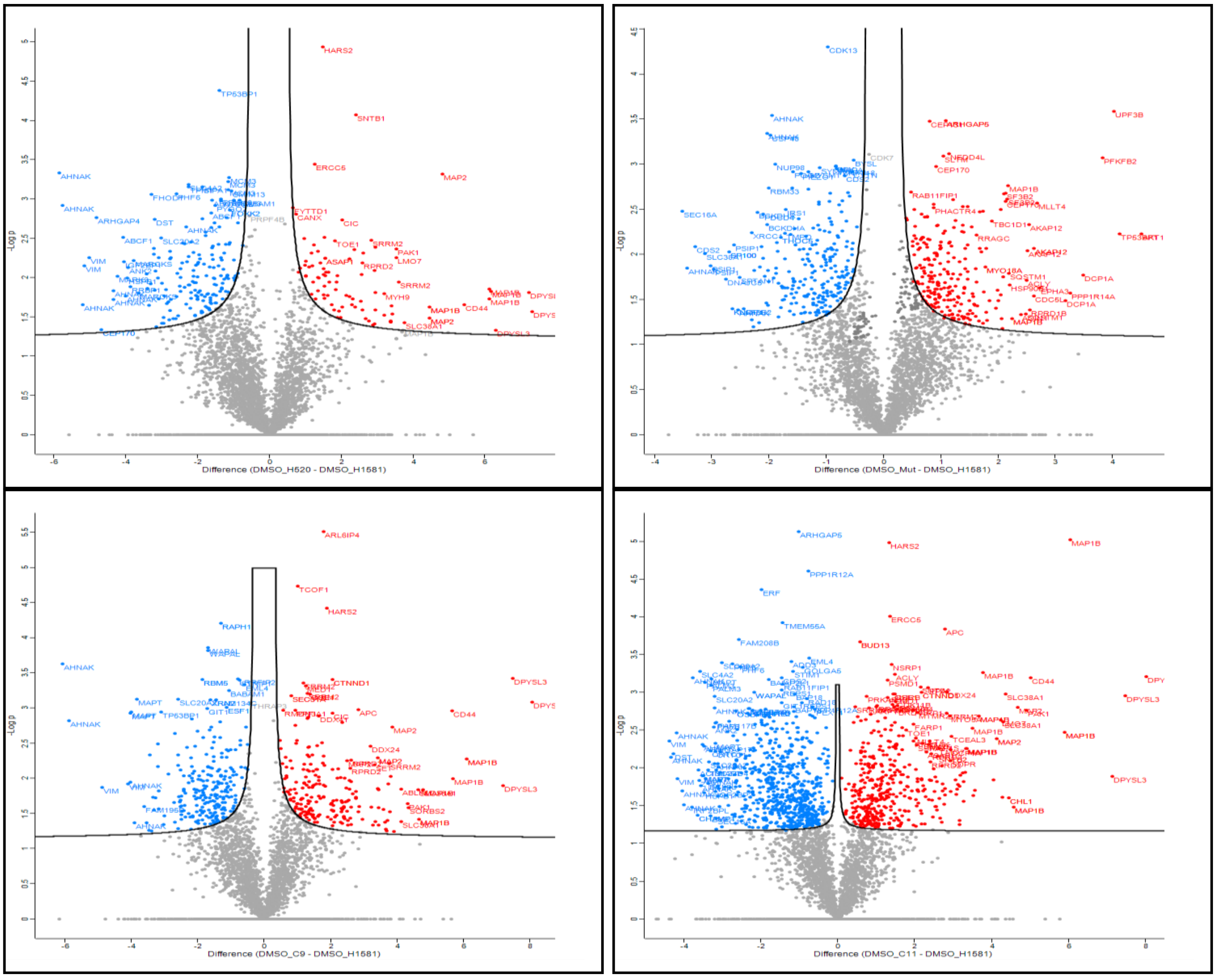

b pYome

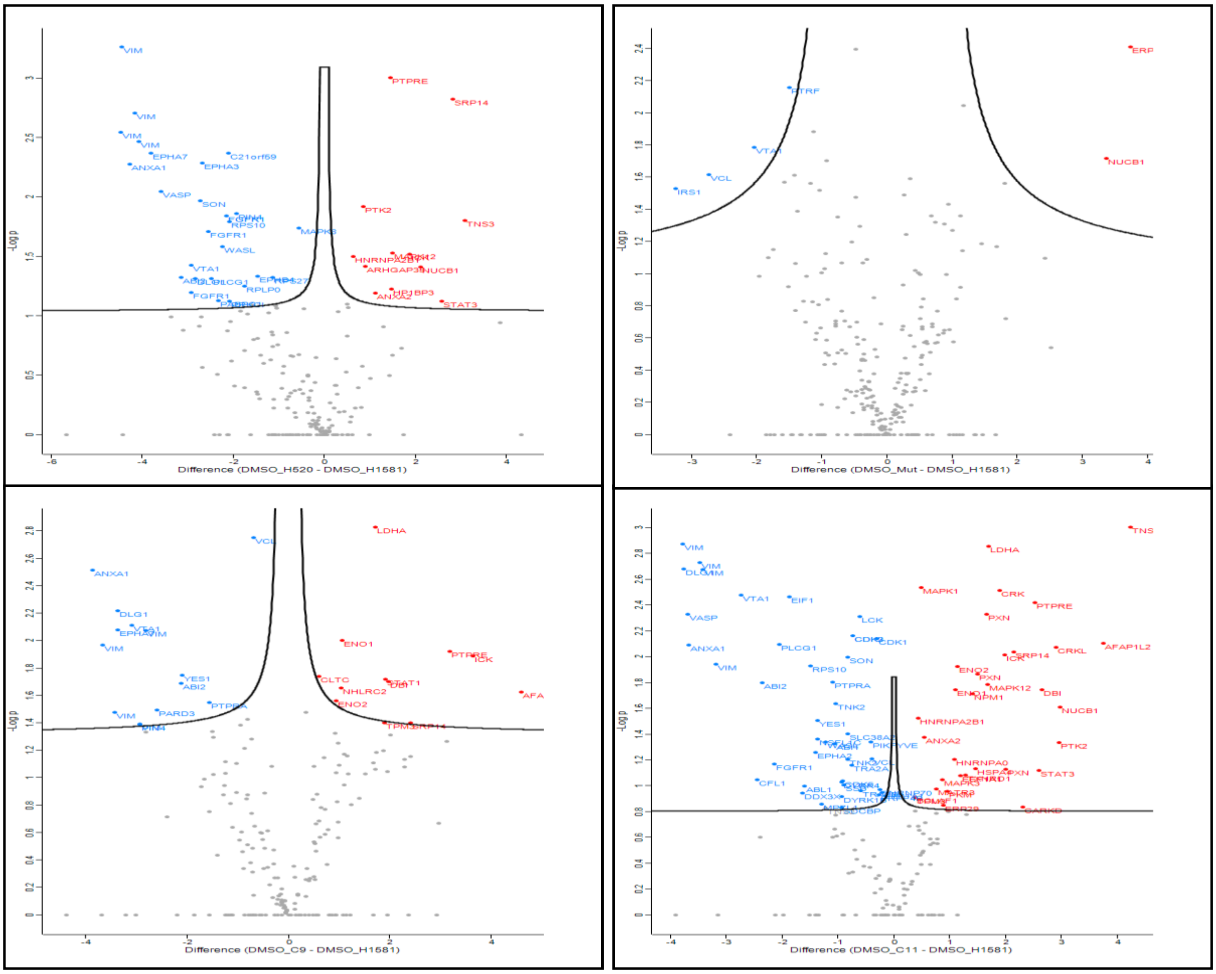

Supplementary Figure 7

c **GPome**

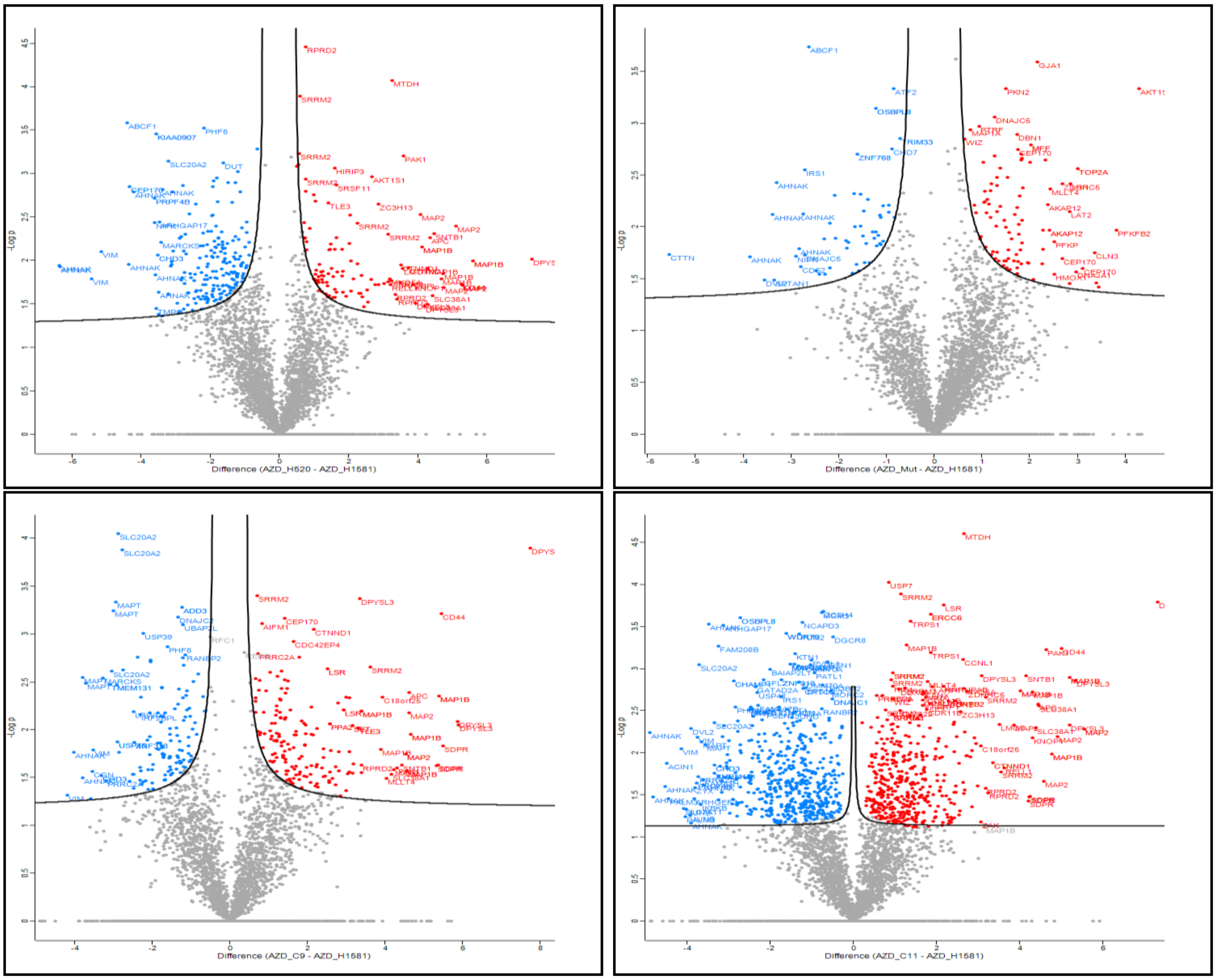

d pYome

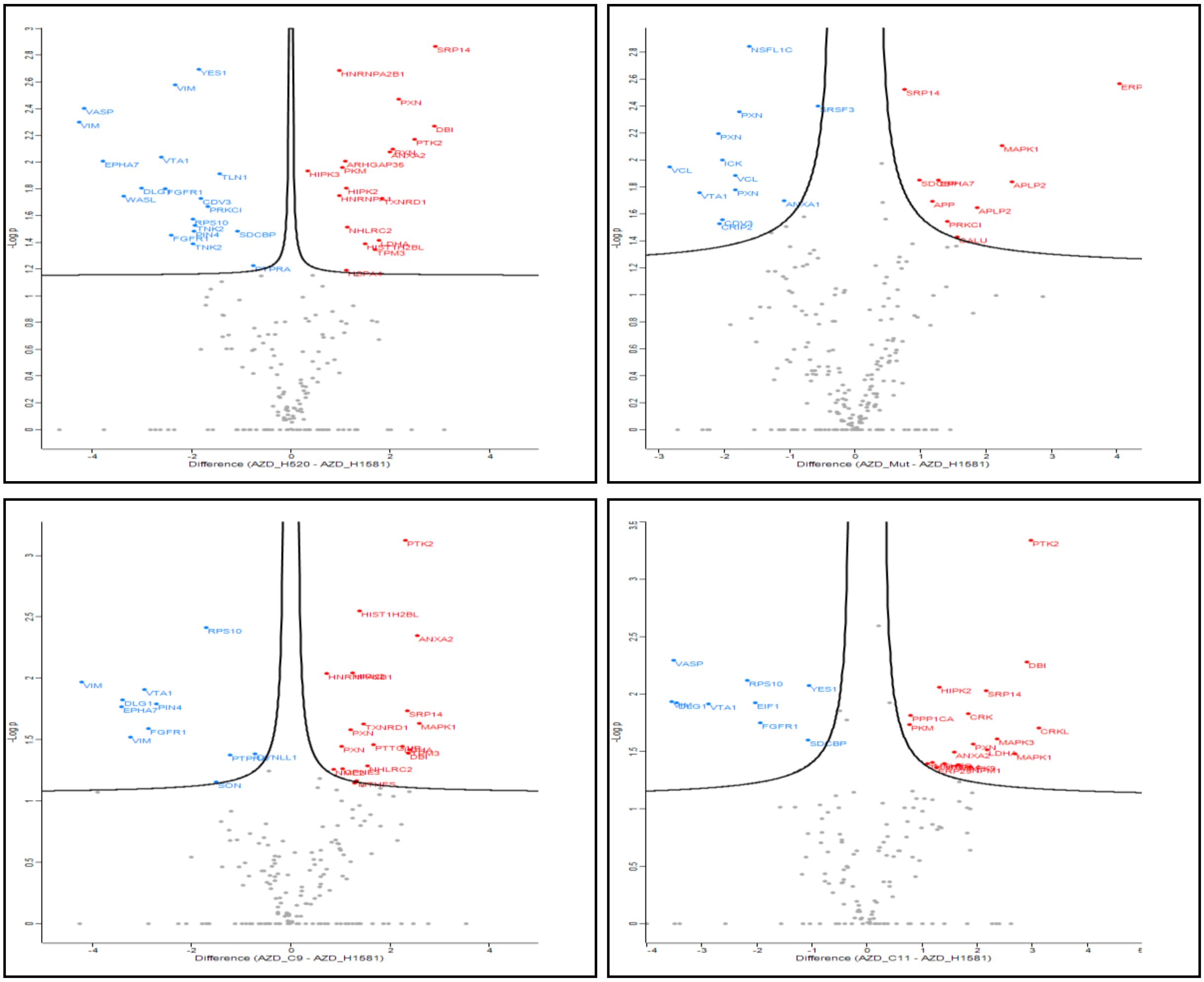

Supplementary Figure 7: Volcano plots show significantly up-/down-regulated phosphosites within the control and FGFR1-resistant cell lines under conditions of DMSO and FGFR1 inhibition.

Supplementary Figure 8

a

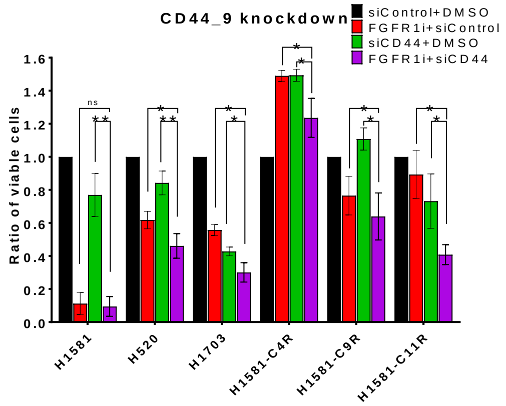

b

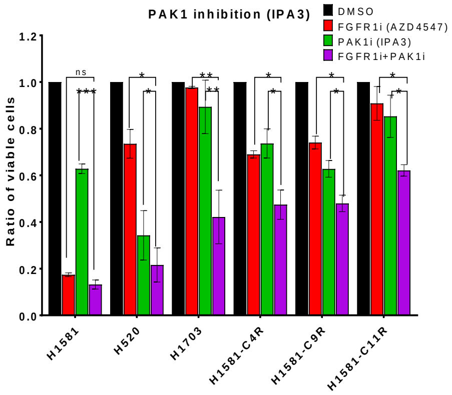

c

Inhibitor (µM)	1	2	3	4	5	6	7	8	9
FGFR1	DMSO	0.03	0.50	1.00	1.20	1.30	1.40	1.50	1.70
FRAX579	DMSO	0.50	0.75	1.00	1.20	1.25	1.30	1.40	1.50
IPA3	DMSO	2.50	10.00	15.00	20.00	25.00	30.00	35.00	40.00

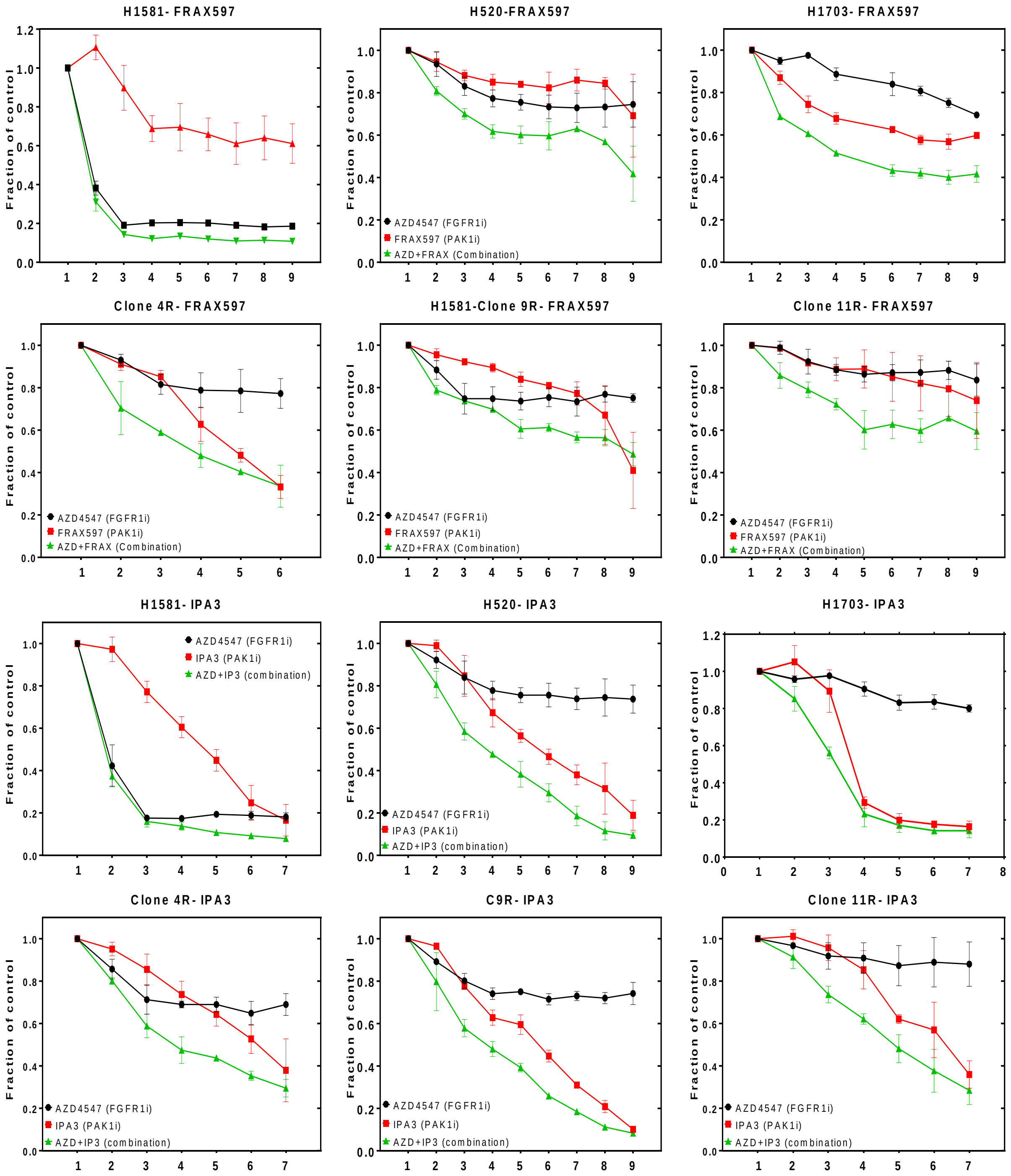

Supplementary Figure 8: (A, B) MTS viability assays show the effect of combining (A) CD44 siRNA knockdown (SI03062661) or (B) PAK1 inhibition (IPA3) with FGFR1 inhibition (AZD4547) in sensitive and resistant cell lines. **(C)** Detailed MTS assay curves of PAK1 inhibition (FRAX597 and IPA) combined with FGFR1 inhibition in control cell lines and cell lines resistant to FGFR1 inhibition. Statistical analysis was performed with the chi-squared test: ns (p>0.05), * (p<0.05), ** (p≤0.01) and *** (p≤0.001). Mean values are plotted; error bars represent standard deviation.

Supplementary Figure 9

Figure 1A
pFGFR1 Y653/654 (92,145 kDa)

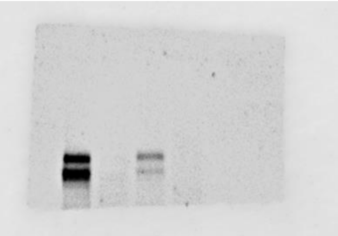

FGFR1 (92,145 kDa)

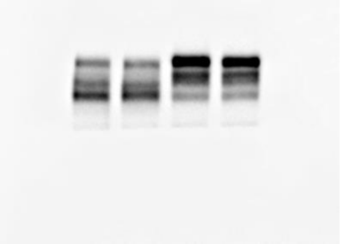

PARK7 (24 kDa)

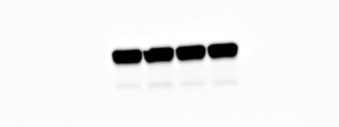

Figure 2A, S2A
pFGFR1 (92,145 kDa) Y653/654

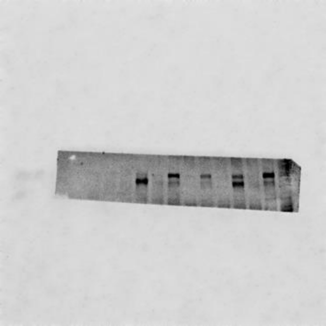

PARK7 (24 kDa)

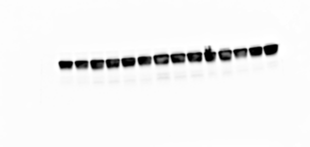

pPRAS40 T246

pAKT (60 kDa) (Ser473

pAKT (60 KDA) Ser473

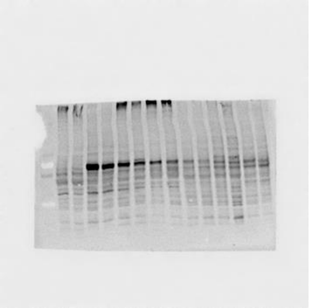

ERK (42, 44 kDa)

pFOXO1 (78 kDa)

pERK (42, 44 kDa)

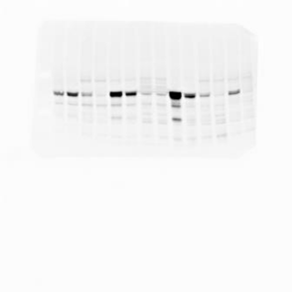

AKT (60 kDa)

FGFR1 (92,145 kDa)

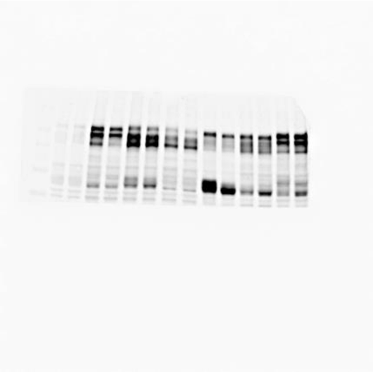

PARK7 (24 kDa)

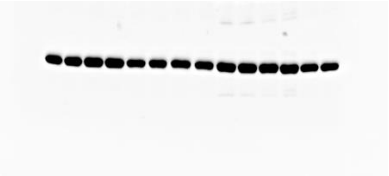

pMEK (45 kDa)

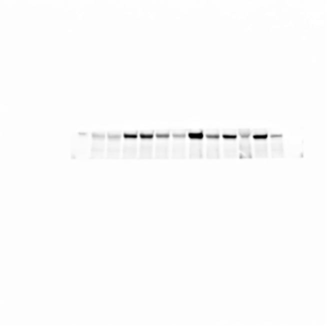

MEK (45 kDa)

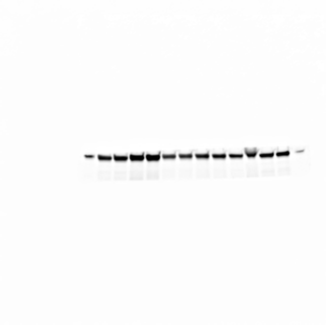

pMET (140, 170 kDa)

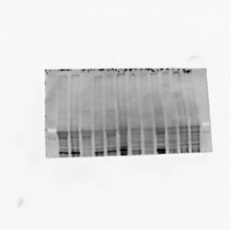

MET (140, 170 kDa)

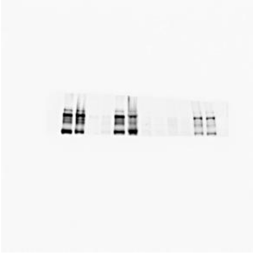

Figure 2C
FGFR1 (92,145 kDa)

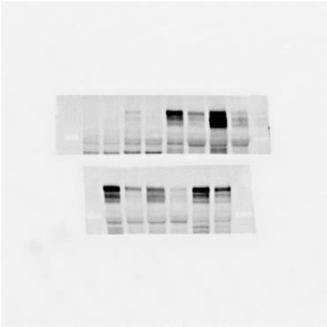

PARK7 (24 kDa)

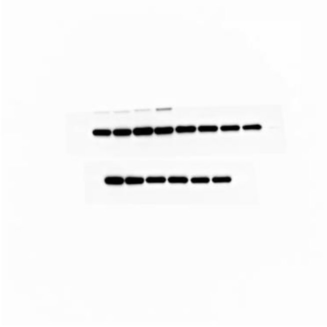

Figure3A, S2E
pAKT (60 kDa) (60 KDA)-Ser

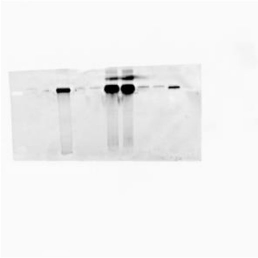

PARK7 (24 kDa)

Figure3E
pAKT (60 kDa) (60 KDA)-T308

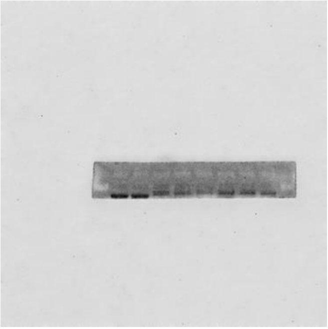

MET (140, 170 kDa)

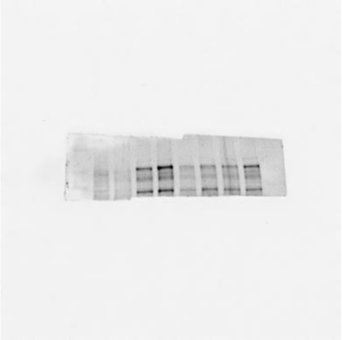

pMET-Y1234/1235

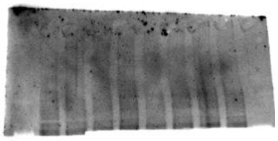

MEK (45 kDa)

ERK (42, 44 kDa)

pAKT (60 kDa) SerS473

pMEK (45 kDa)

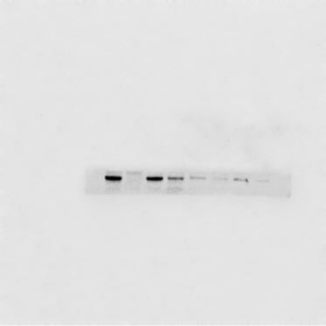

FGFR1 (92,145 kDa)

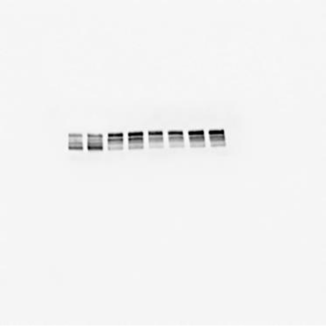

Figure 5B
CD44 (80 kDa)

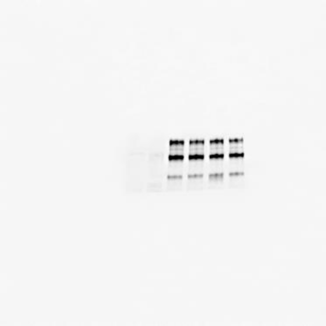

PARK7 (24 kDa)

pFGFR1 (92,145 kDa)
Y653/Y654

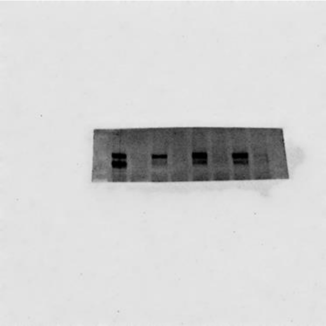

PARK7 (24 kDa)

pPRAS40 T246

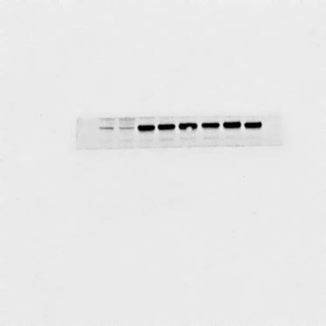

pERK (42, 44 kDa)
T202/Y204

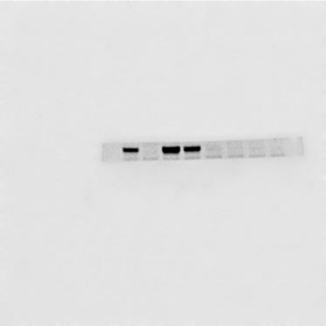

pAKT (60 kDa) Ser473

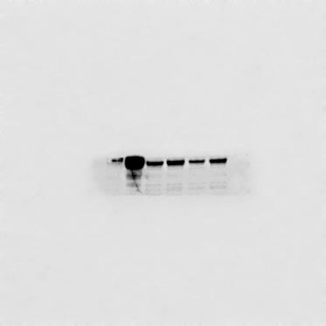

pCD44 (80 kDa) S706

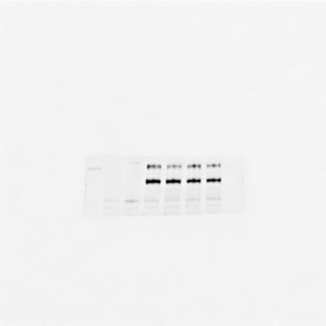

AKT (60 kDa)

pPRAS40 T246 (40 kDa)

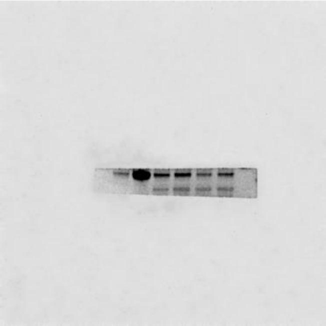

Figure 5C
pCD44 (80 kDa) S706
H1581-C4R

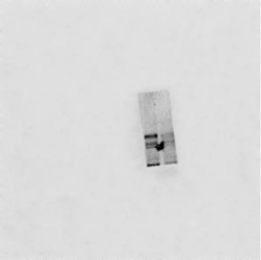

pCD44 (80 kDa) S706
H520

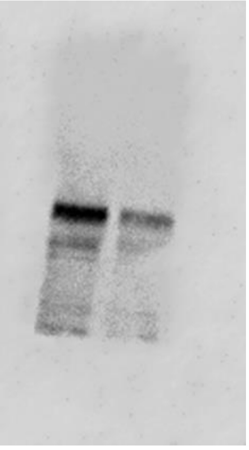

pCD44 (80 kDa) S706
H1581-C9R

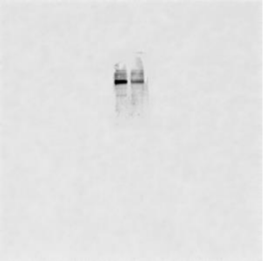

pCD44 (80 kDa) S706
H1581-C11R

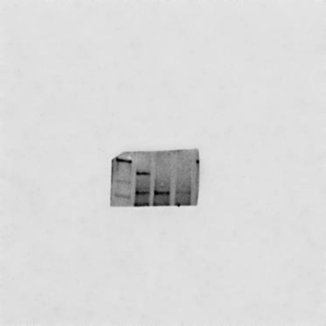

PAK1 (68 kDa) H1581-C4R

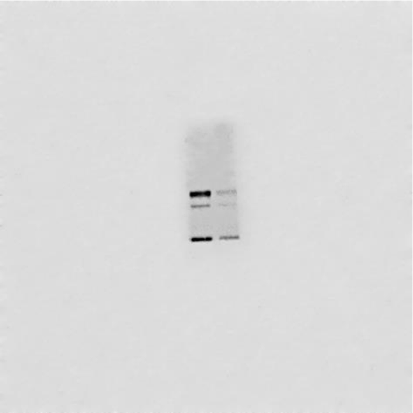

PAK1 (68 kDa) H520

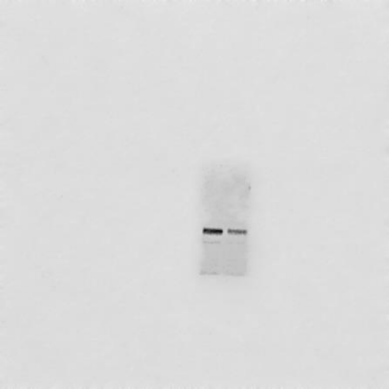

PAK1 (68 kDa) H1581-C9R

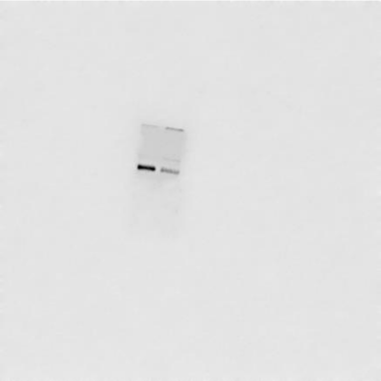

PAK1 (68 kDa) H1581-C11R

pPAK1 (68 kDa) S144 H1581-C4R

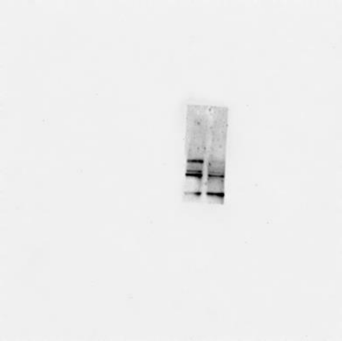

pPAK1 (68 kDa) S144 H1581-C9R

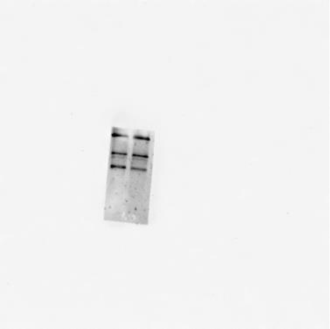

pPAK1 (68 kDa) S144 H520

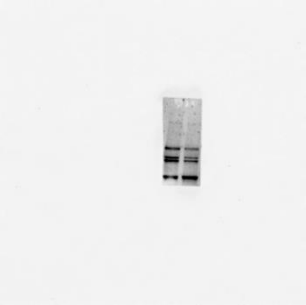

pPAK1 (68 kDa) S144 H1581-C11R

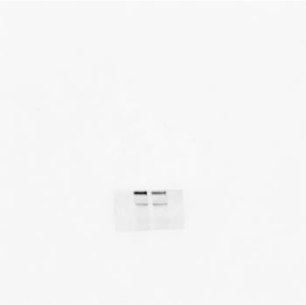

PARK7 (24 kDa) H1581-C4R

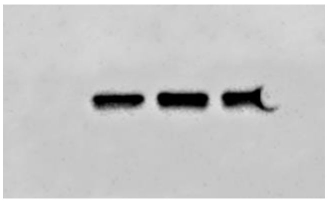

PARK7 (24 kDa) H1581-C9R

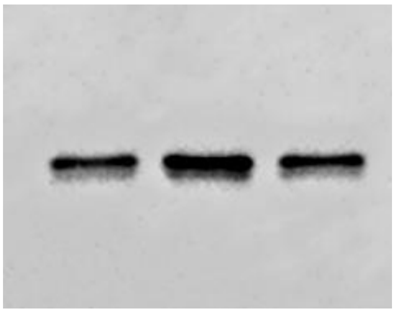

PARK7 (24 kDa) H1581-C11R

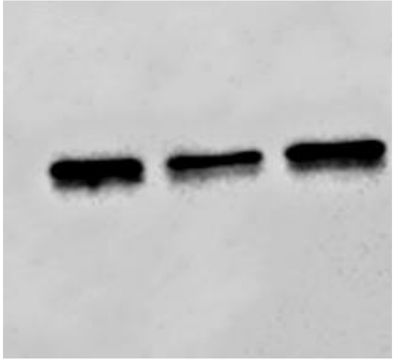

PARK7 (24 kDa) H520

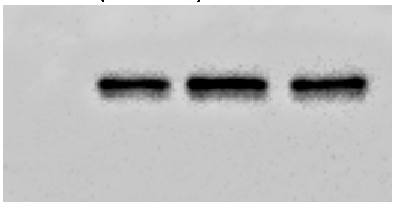

CD44 (80 kDa) H1581-C11R

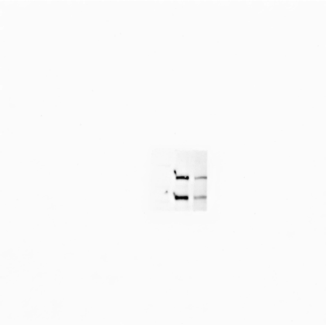

CD44 (80 kDa) H1581-C4R

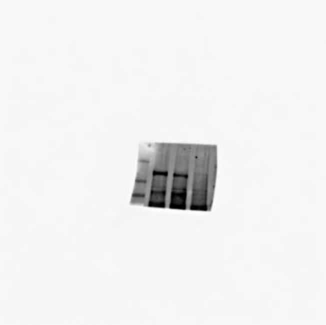

CD44 (80 kDa) H1581-C9R

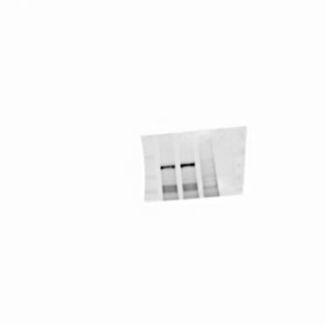

CD44 (80 kDa) H520

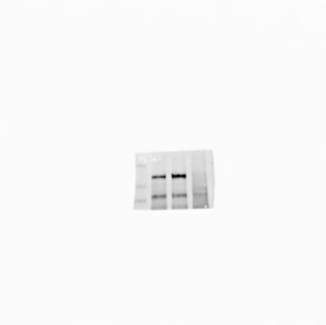

pAKT (60 kDa) Ser473
H1581-C4R

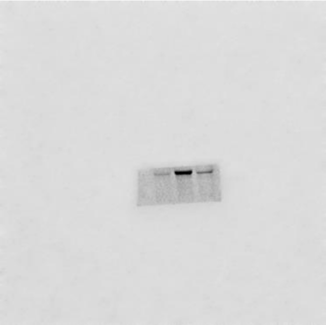

pAKT (60 kDa) Ser473
H1581-C9R

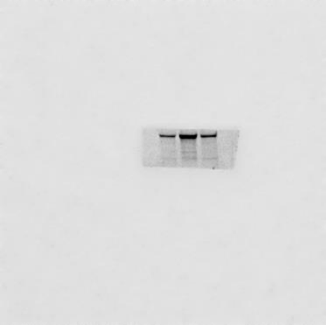

pAKT (60 kDa) Ser473
H1581-C11R

pAKT (60 kDa) (60 KDA)
Ser473 H520

AKT (60 kDa) H1581-C11R

AKT (60 kDa) H520

AKT (60 kDa) H1581-C4R

AKT (60 kDa) (60 KDA)
H1581-C9R

Figure 5D
AKT (60 kDa)

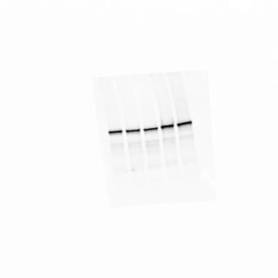

PAK1 (68 kDa)

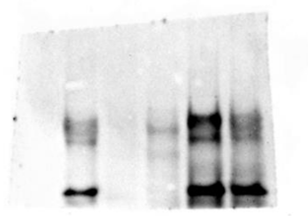

pPAK1 (68 kDa) S144

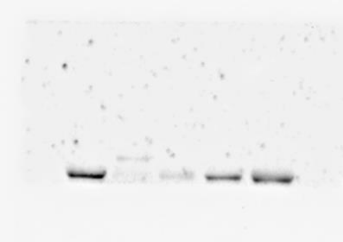

pAKT (60 kDa) (60 KDA)
Ser473

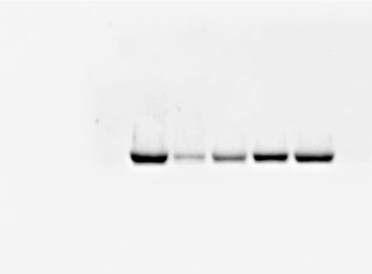

PARK7 (24 kDa)

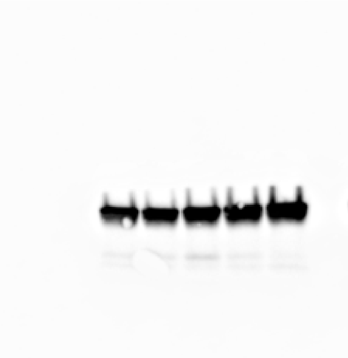

pPRAS40 T246 (40 kDa)

pCD44 (80 kDa) (80 kDa)
S706

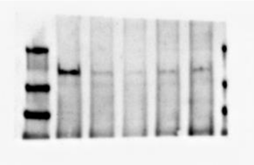

CD44 (80 kDa)

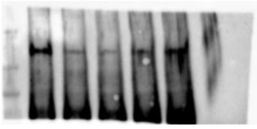

Figure 5E
pCD44 (80 kDaS706

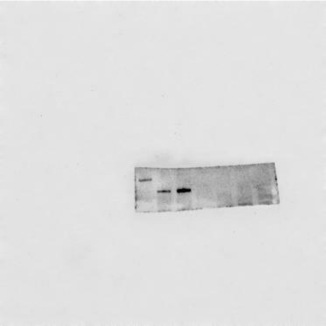

CD44 (80 kDa)

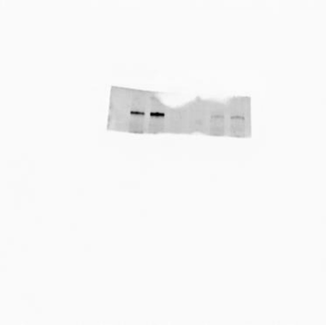

pAKT (60 kDa) Ser473

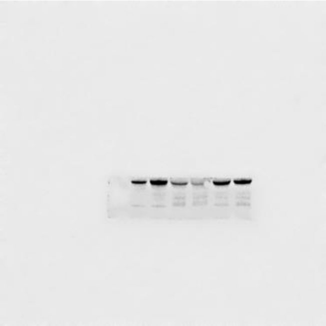

AKT (60 kDa)

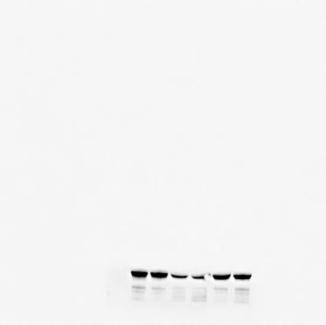

PARK7 (24 kDa)

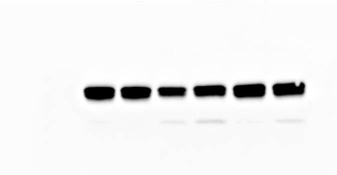

pPRAS40 T246

PAK1 (68 kDa)

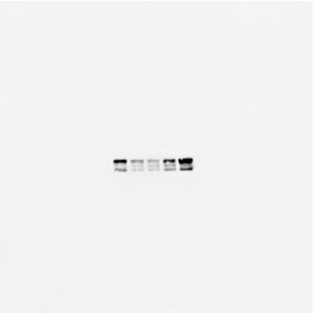

pPAK1 (68 kDa) S144

FIGURE 5H
PAK1 (68 kDa) H1581-C9R

PAK1 (68 kDa) H520

pPAK1 (68 kDa) S144
H520

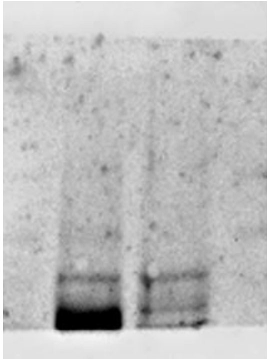

pPAK1 (68 kDa) S144 H1581-C9R

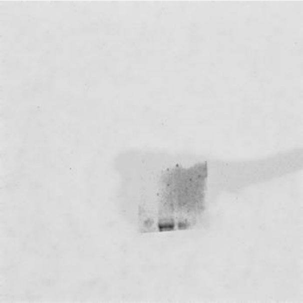

AKT (60 kDa) H520

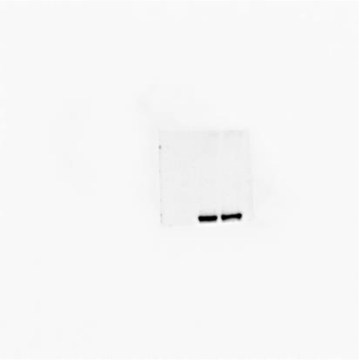

pAKT (60 kDa) Ser473 H520

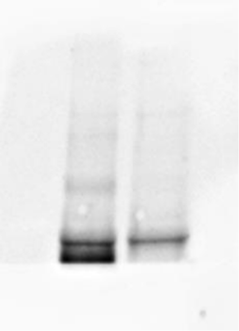

pAKT (60 kDa) (60 KDA) S473 H1581-C9R

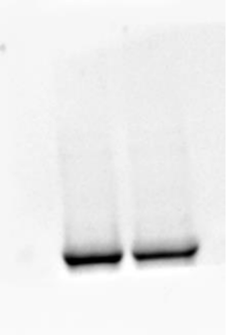

pPRAS40 T246 H520 (40 kDa)

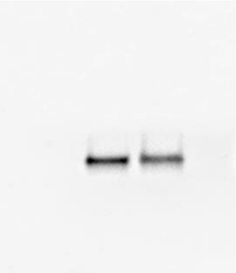

pPRAS40 T246 H1581-C9R (40 kDa)

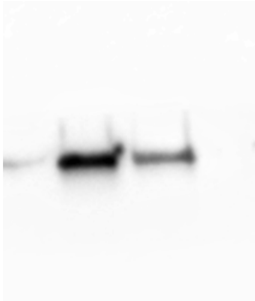

PARK7 (24 kDa) H520

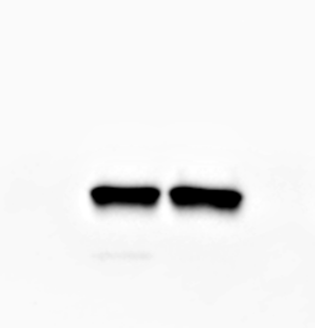

Supplementary Figure2B pPRAS40 T246 (40 kDa)

pAKT (60 kDa) Ser473

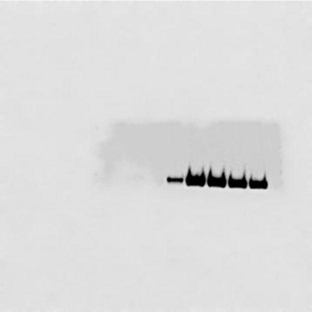

PARK7 (24 kDa)

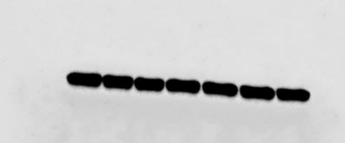

Supplementary Figure 3A pAKT (60 kDa) Ser473 H1581

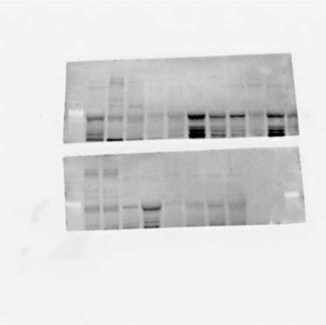

pAKT (60 kDa) Ser473 DMS114

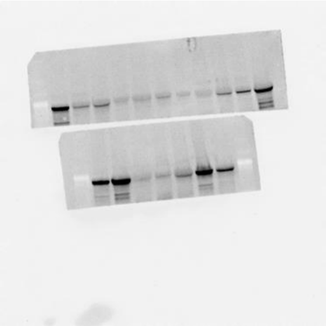

pAKT (60 kDa) Ser473 LK2

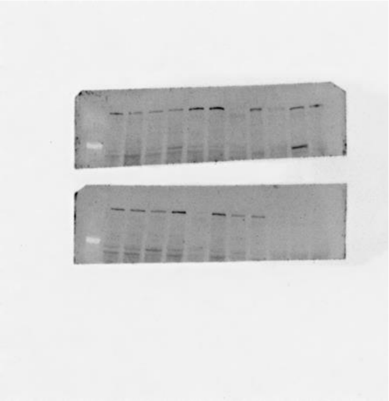

PARK7 (24 kDa) H1581

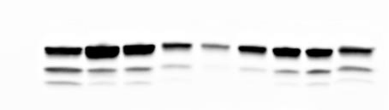

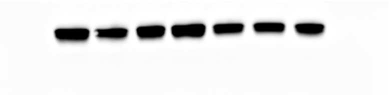

PARK7 (24 kDa) DMS114

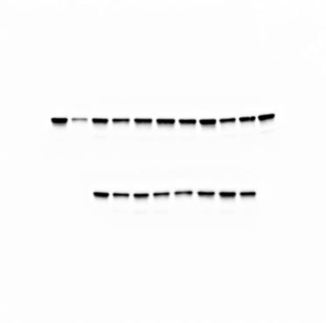

PARK7 (24 kDa) LK2

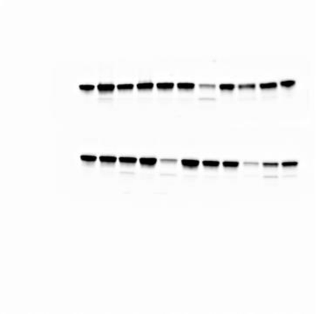

Supplementary Figure 3B

pPRAS40 T246 (40 kDa)

pAKT (60 kDa) Ser473

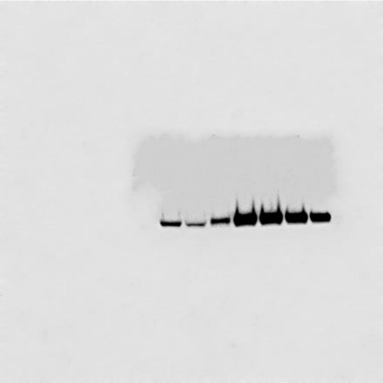

PARK7 (24 kDa)

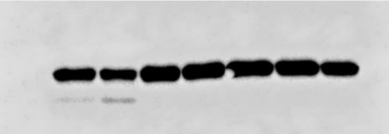

Supplement: Supplementary file 1 — Supplementary_Information [file 41698_2022_296_MOESM1_ESM.pdf]
